# Evaluating long-term programmatic outcomes of patients with advanced HIV disease: Insights from 12 years of antiretroviral therapy scale-up in rural KwaZulu-Natal

**DOI:** 10.1371/journal.pone.0360657

**Published:** 2026-10-27

**Authors:** Marcel K. Kitenge, Geoffrey Fatti, Chinmay Laxmeshwar, Patrick D. M. C. Katoto, Peter S. Nyasulu

**Affiliations:** 1 Division of Epidemiology and Biostatistics, Department of Global Health, Faculty of Medicine and Health Sciences, Stellenbosch University, Cape Town, South Africa; 2 School of Mathematics, Statistics & Computer Science, University of KwaZulu-Natal, Durban, South Africa; 3 Department of Global Health, University of Washington, Seattle, Washington, United States of America; 4 Centre for Tropical Diseases and Global Health, Department of Medicine, Catholic University of Bukavu, Bukavu, DR Congo; 5 Division of Epidemiology and Biostatistics, School of Public Health, Faculty of Health Sciences, University of the Witwatersrand, Johannesburg, South Africa; London School of Hygiene and Tropical Medicine, UNITED KINGDOM OF GREAT BRITAIN AND NORTHERN IRELAND

## Abstract

**Background:**

Advanced HIV disease (AHD) remains a significant public health challenge, despite the widespread scale-up of antiretroviral therapy (ART) in South Africa. Understanding long-term treatment outcomes among patients with AHD under routine programmatic conditions is critical. However, there is a paucity of longitudinal data on clinical outcomes in this population, particularly within high-HIV prevalence rural settings. This study evaluated retention in care and virological outcomes among patients initiating ART with AHD in rural KwaZulu-Natal between 2008 and 2020.

**Methods:**

We conducted a retrospective cohort study using routine data from 10 public health facilities. Adults (≥18 years) with CD4 < 200 cells/μL or WHO stage 3/4 who initiated ART between January 1, 2008, and December 31, 2020, were included. Primary outcomes were retention in care at 6, 12, 24, 36, and 48 months. Secondary outcomes included viral load (VL) completion and suppression (<1000 copies/μL). Competing risk regression and Poisson regression models were used to identify factors associated with loss to follow-up (LTFU) and virological suppression.

**Results:**

Of 40,432 individuals who initiated ART between 2008 and 2020, 9,608 met the criteria for AHD. Retention in care was 98%, 94%, 89%, and 85% at 12, 24, 36, and 48 months, respectively. Men (adjusted subdistribution hazard ratio [aSHR]: 1.35; 95% confidence interval [CI]: 1.23–1.47) and individuals with CD4 counts <100 cells/µL (aSHR: 1.13; 95% CI: 1.08–1.26) had a higher risk of loss to follow-up. Although viral load (VL) completion declined over time (49.3% at 6 months to 31.5% at 48 months), suppression remained above 83% among those with available VL data. Undetectable VL increased from 25.5% to 33.0% between 12 and 48 months. Patients initiating ART during the Universal Test and Treat (UTT) era (2016–2020) had higher retention (78.8%) than those treated between 2012–2015 (62.8%) and higher viral load (VL) suppression (risk ratio [RR]: 1.13; 95% CI: 1.01–1.28) compared with the 2008–2011 cohort. Women (RR 1.18; 95% CI: 1.11–1.25) and those initiating ART within 7 days (RR 1.30; 95% CI: 1.04–1.38) were more likely to achieve virological suppression at any time point.

**Conclusion:**

This study demonstrates high retention and virological suppression rates among patients with AHD in rural South Africa, particularly under the UTT strategy. Despite these successes, VL testing uptake remains suboptimal, and a considerable gap persists in achieving undetectable VL levels. Integrating the WHO AHD care package and prioritizing early ART initiation and male-focused interventions may further improve outcomes and support efforts to end the HIV epidemic.

## 1. Background

The global community has set ambitious targets for the rollout and success rates of antiretroviral treatment (ART) to end the HIV epidemic by 2030. These 95-95-95 targets, set by the Joint United Nations Program on HIV/AIDS (UNAIDS), aim to ensure that by 2025, 95% of people living with HIV (PLWH) receiving ART achieve viral load suppression (HIV-RNA suppression) [1]. While progress towards the UNAIDS 95-95-95 targets has been generally slower in low-and middle-income countries [2], South Africa is estimated to have expanded access to antiretroviral therapy (ART) from approximately 103,300 people living with HIV (PLWH) in 2005 to an estimated 5,936,502 by 2023 [3]. However, despite these advancements, the burden of Advanced HIV Disease (AHD) in South Africa remains persistently high [4–7]. The WHO defines AHD as CD4 count of fewer than 200 cells/μL or WHO stage 3 or 4 event in adults and adolescents, and all children with HIV younger than five years old [8]. In 2023, an approximately 33% of PLWH presenting to HIV services in South Africa were estimated to be presening in care with AHD at ART, with notable variation across gender and age groups [9]. Furthermore, individuals presenting with AHD in HIV services are often not prioritized, further complicating efforts to improve treatment outcomes [10].

Patients with AHD are at increased risks of mortality, even after initiating ART, as well as at a higher risk of opportunistic infections and greater treatment costs compared to PLWH without AHD [11,12]. While previous changes to ART guidelines aimed at increasing the treatment eligibility threshold have produced mixed results [13–15], they have generally led to better ART coverage among rural areas [16]. Several cross-sectional, population-based surveys conducted in South Africa have shown that rural settings performed better on the UNAIDS 95-95-95 treatment targets than urban settings [17–19]. However, rural PLWH still face greater barriers to HIV care compared to their urban counterparts [19–21]. Detailed data on long-term treatment outcomes under routine programmatic conditions among patients with AHD remain limited in rural South Africa, particularly in high-HIV-prevalence, low-income settings.

To achieve the Global AIDS Strategy 2026–2031 target of reducing AIDS-related deaths by 90% by 2030, compared with 2010 levels [1] and to inform local policies and programs, it is crucial to better characterize and assess long-term treatment outcomes of patients with AHD under routine programmatic conditions, particularly in HIV high-prevalence, low-income rural settings. To address this gap, we evaluated clinical outcomes, including retention in care and viral load suppression, in a cohort of patients with AHD receiving ART in rural KwaZulu-Natal, South Africa.

## 2. Methods

### 2.1. Design

This retrospective cohort study included adults who initiated ART with a CD4 < 200 cells/μL or AHD between January 1, 2008, and December 31, 2020, using routinely collected ART program data at 10 public health facilities.

### 2.2. Participants and settings

We included patients aged 18 years and older with AHD at ART initiation to a health facility who initiated ART at 10 public primary care clinics in Eshowe and Mbongolwane areas, KwaZulu-Natal, South Africa.

According to a population-based survey conducted by MSF, these areas exceeded the UNAIDS 90-90-90 targets by achieving 90-94-95 in 2018. The survey also revealed that HIV prevalence in the study areas was an estimated 26.4 among 15–59 years old adults [19]. This region is primarily rural, with one semi-urban market town, and had a combined population of 114,490 in 2021 [22].

Throughout the analysis period, South African ART guidelines changed starting with eligibility for ART initiation was determined by low CD4 threshold: < 200 cells/µl up to 2010 [23] and <350 cells/µl between 2011–2013 [24,25]. In January 2015, South Africa removed CD4 eligibility criteria for pregnant or breastfeeding women (Option B+) and expanded CD4 eligibility to ≤500 cells/µl for nonpregnant or nonbreastfeeding women and all adult men [26]. Lastly, in September 2016, South Africa implemented the Universal Test and Treat (UTT) strategy, allowing for ART initiation regardless of CD4 count level or clinical stage of HIV disease [10,27,28].

### 2.3. Data source and variables

We used de-identified data from South Africa’s TIER.NET, an electronic database used in public health facilities for the management of HIV and tuberculosis (TB). TIER.NET contains demographic and clinical data of PLWH receiving ART and TB care in public sector healthcare clinics. The information captured includes ART and regimens, laboratory results (CD4 count, HIV viral load), clinic visits, and TB status at ART initiation and each follow-up visit [29]. For this analysis, data on clients initiating ART in the Eshowe and Mbongolwane areas of KwaZulu-Natal were extracted from Tier.Net on 31 May 2022 by the District Health Information Systems Manager and provided to the study authors.

### 2.4. Outcomes and definitions

The primary outcome of interest was retention in care at 6, 12, 24, 36, and 48 months after ART initiation. Retention was defined as the time from ART initiation to lost to follow-up (LTFU), death and transferred out. Individuals who remained on treatment at the end of the 48-month follow-up period were considered censored. Patients were considered as LTFU if they had not returned to care 90 days after their scheduled appointment.

We had two secondary outcomes: viral load (VL) completion and VL suppression among those with a completed VL test at 6, 12, 24, 36, and 48 months. VL completion was defined as having an annual monitoring VL test, as per Department of Health (DoH) guidelines [30]. To estimate VL completion, we constructed a patient-specific window period based on the ART initiation date, within which the VL test would have been due. This VL testing window allowed a margin of ±3 months around the expected VL due date [31]. For example, 12-month VL completion is assessed for all patients who have been followed up for at least 15 months and have had a VL test conducted between 9 and <15 months after initiating ART. If multiple VL measurements were taken within the annual window period, the latest result is used. VL suppression was defined as a plasma VL of <1,000 copies/μL. Additionally, patients with a plasma VL of <50 copies/μL are classified as having an undetectable VL.

### 2.5. Statistical analysis

Continuous variables were summarised using mean and standard deviation (SD) if normally distributed, otherwise median, and interquartile range (IQR) were used, and categorical variables were summarised using percentages. Baseline characteristics and programmatic outcomes were described with frequencies and proportions and compared using Pearson’s chi-squared test for categorical variables. For time-to-event analyses, follow-up was calculated in months from ART initiation to the last clinic visit for patients who were known to have died, transferred out, or were LTFU. Follow-up was censored at 48 months after ART initiation for those without these outcomes. To prevent zero follow-up time among patients who did not return to the health facility after enrollment, one month of follow-up time was added for all patients. Kaplan-Meier estimates were used to describe retention in care, VL completion, VL suppression, and undetectable VL.

Time-to-LTFU was evaluated by competing risk (CR) analysis using cumulative incidence function, with death as a competing event. A CR analysis assumes that patients were exposed to at least two competing events (LTFU or death). The event of interest is LTFU, while the others are competing events that prevent the outcome of interest from occurring [32–34]. We used Fine-Gray competing risks regression with a proportional sub-distribution hazard (SHR) model adjusted for clustering at the facility to model cumulative incidence, quantify the instantaneous risk of failure from LTFU, and assess the overall effect of covariates on the cumulative incidence of LTFU, treating death as a competing risk [32,35]. Additionally, cumulative event graphs were used to assess differences between sex, CD4 count groups, TB status, ART initiation periods, and time-to-LTFU

log-binomial regression model was used to study the association between demographic and clinical variables and having ever achieved viral load suppression. The adjusted risk ratio (RR) was used as the measure of association. Multivariable models were adjusted for sex, age, and baseline CD4 count at care entry. Measures of associations and proportions were presented with 95% confidence intervals (CI) and factors with p < 0.05 were considered statistically significant. All analyses were performed with STATA 18.1 (StataCorp, College Station, Texas, USA).

### 2.6. Ethical statement

The study was approved by the Health Research Ethics Committee (HREC) at Stellenbosch University (S21/11/253) and the KwaZulu-Natal Department of Health’s Provincial Health Research Ethics Committee (NHRD Ref: KZ_202204_018). Waiver of consent was obtained from the ethics committee for use of retrospective data for this study.

## 3. Results

### 3.1. Patient characteristics

Fig 1 shows the study flow of our analysis. Among 40,432 PLHIV who initiated ART between January 01, 2008, and December 31, 2020, 9608 (23.7%) met the definition for AHD. Of these 3,420 (35.6%) initiated ART during the 2008–2011 period, 3,761 (39.1%) initiated ART during 2012 and 2015, and 2,427 (49.3%) initiated during the Universal Test and Treat (UTT) (2016–2020). Table 1 shows demographic characteristics of patients included in the analysis by period of ART initiation. The mean age at ART initiation was 36.1 years (SD: 10.1 years), the median CD4 count was 112.0 cells/μL (Interquartile range [IQR] 58–159), and median follow-up was 29 months (IQR 6–77). Additionally, 6.6% of patients had TB at the time of ART initiation. Under UTT era, 21% of patients with AHD who initiated ART had TB, compared-only 2% between 2012 and 2015 (p < 0.001). Of those who initiated ART, 17.0% initiated within 7 days, while 83.0% initiated ART after 8 days or more. There was a statistically significant difference in the proportion of patients with AHD who initiated ART within 7 days across different time periods: 15 (0.5%) during 2008–2011, 1,186 (31.5%) during 2011–2015, and 423 (17.4%) during 2016–2020 (p < 0.001). Majority of patients were on a TDF + FTC/3TC+EFV-based regimen. All patients who met the inclusion criteria, 44.1% had a CD4 0–99 cells/μL and 55.9% had a CD4 count of 100–199 cells/μL.

**Table 1:** Baseline characteristics of patients with advanced HIV disease at ART initiation.

Variables	All	2008-2011	2012-2015	2016-2020	p-values
	**N = 9,608**	**N = 3,420**	**N = 3,761**	**N = 2,427**	
**Sex n (%)**					
Women	5,481 (57.0)	2,161 (63.2)	2,116 (56.3)	1,204 (49.6)	<0.001
Men	4,127 (43.0)	1,259 (36.8)	1,645 (43.7)	1,223 (50.4)	
**Age at HIV care**					
18-24	907 (9.4)	252 (7.4)	424 (11.3)	229 (9.4)	<0.001
25-31	2,668 (27.8)	891 (26.1)	1,113 (29.6)	664 (27.4)	
32-38	2,676 (27.8)	928 (27.1)	1,019 (27.1)	729 (30.0)	
39-45	1,710 (17.8)	676 (19.8)	622 (16.5)	412 (17.0)	
>45	1,647 (17.1)	671 (19.6)	583 (15.5)	393 (16.2)	
**Baseline CD4 counts**					
0-99 cells/μL	4,238 (44.1)	1,411 (41.3)	1,693 (45.0)	1,134 (46.7)	<0.001
100-199 cells/μL	5,370 (55.9)	2,009 (58.7)	2,068 (55.0)	1,293 (53.3)	
**Tuberculosis**					
No	8,978 (93.4)	3,380 (98.8)	3,686 (98.0)	1,912 (78.8)	<0.001
Yes	630 (6.6)	40 (1.2)	75 (2.0)	515 (21.2)	
**WHO Stage**					
1	1,717 (17.9)	136 (4.0)	642 (17.0)	941 (39.0)	<0.001
2	1,594 (16.6)	235 (6.9)	798 (21.2)	561 (23.1)	
3	2,600 (27.0)	675 (19.7)	1,484 (39.4)	441 (18.1)	
4	379 (4.0)	79 (2.3)	194 (5.2)	106 (4.3)	
Not ascertained	3,316 (34.5)	2,295 (67.1)	643 (17.1)	378 (15.5)	
**ART regimen on enrolment**					
ABC + 3TC	58 (0.6)	1 (0.1)	19 (0.5)	38 (1.6)	<0.001
AZT + 3TC	138 (1.5)	66 (2.0)	63 (1.7)	9 (0.4)	
TDF + FTC/3TC + EFV	7,334 (76.3)	1,459 (42.6)	3,583 (95.2)	2,292 (94.4)	
Missing	2,078 (21.6)	1,894 (55.4)	96 (2.5)	88 (3.6)	
**Time-ART Initiation**					
1-7 days	1,624 (17.0)	15 (0.4)	1,186 (31.5)	423 (17.4)	
8 days and more	7,984 (83.0)	3,405 (99.6)	2,575 (68.5)	2,004 (82.6)	<0.001

ART: antiretroviral therapy; ABC; Abacavir; AZT, Zidovudine; EFV, efavirenz; 3TC, Lamivudine; TDF, Tenofovir; FTC, Emtricitabine

**Fig 1:**
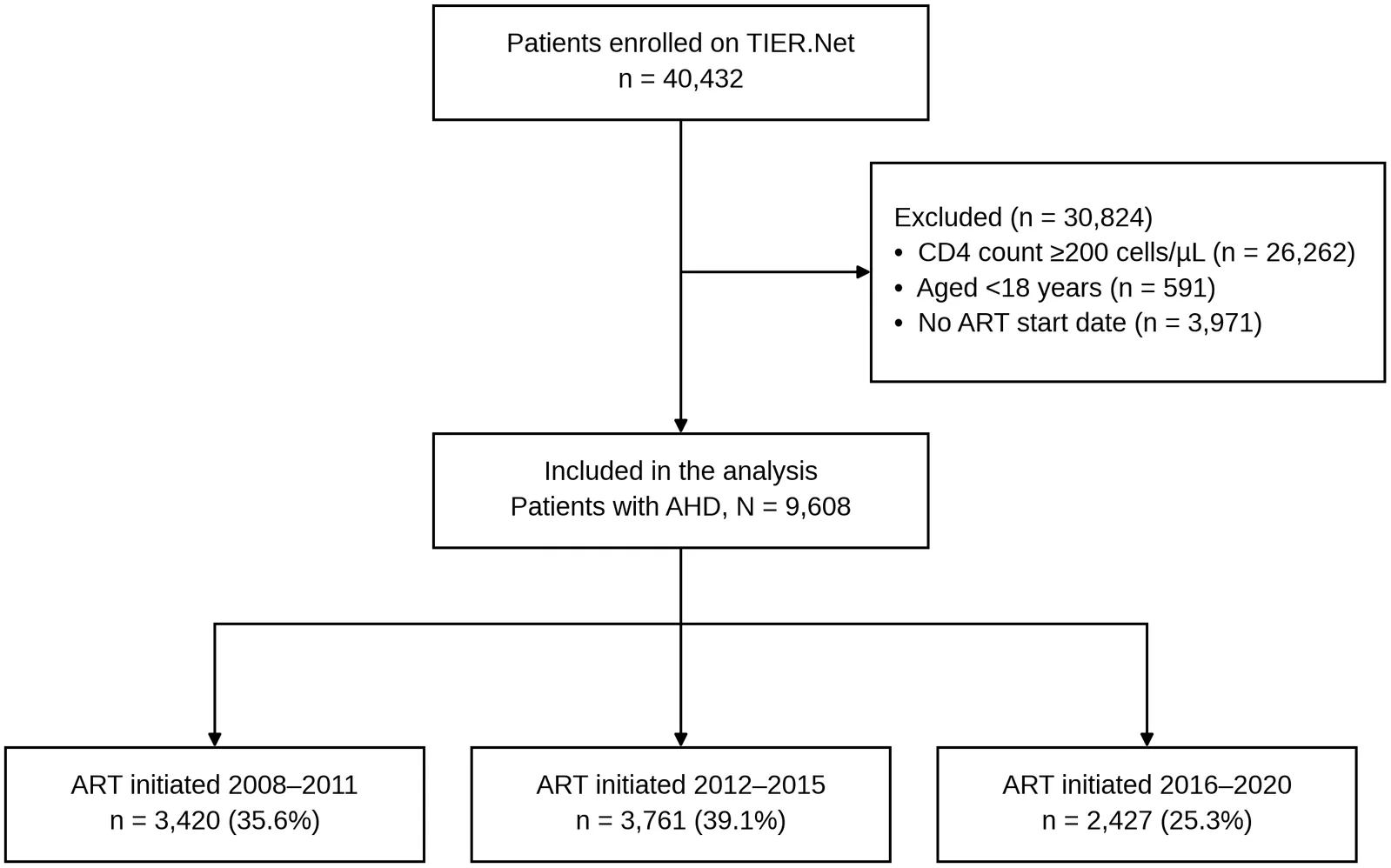
Study flow, overview of patients included in the analysis.

### 3.2. HIV Programmatic outcomes

Of the 9,608 patients included in the study, over the observation period, the majority, 6,551 (68.2%), remained in care at 48 months, while 2,428 (25.2%) were LTFU and 639 (6.7%) died. Retention in care at 48 months was significantly higher among those who initiated ART between 2016–2020 (UTT era) compared to earlier periods (p < 0.001) and mortality rates was significantly lower among individuals who initiated ART during **2016–2020 (p < 0.001)** (Table 2).

**Table 2:** Outcomes among patients with AHD at 48 months, by period of ART initiation.

Outcomes	All N = 9,608	2008-2011 N = 3,420	2012-2015 N = 3,761	2016-2020 N = 2,427	p-value
Active in care	6,551 (68.2%)	2,280 (66.7%)	2,359 (62.8%)	1,912 (78.8%)	<0.001
LTFU	2,428 (25.2%)	870 (25.4%)	1,139 (30.2%)	409 (16.8%)	
Died	639 (6.7%)	270 (7.9%)	263 (7.0%)	106 (4.4%)	

### 3.3. Retention in care among patients with AHD

The cumulative number of person-years of observation for the cohort that contributed to surviving cases was 40,779.7 person-years. The median time from ART initiation to LTFU was 35.2 months (IQR 6.3–165.2). At 12 months, the incidence rate of LTFU was 0.21 (95% CI 0.17–0.26) cases per 100 person-years. At 24 months, it increased to 0.36 (95% CI 0.32–0.41) cases per 100 person-years. At 36 months, the incidence rate was 0.49 (95% CI 0.44–0.55) cases per 100 person-years, before slightly decreasing to 0.42 (95% CI 0.36–0.48) cases per 100 person-years at 48 months.

Crude retention was 98% (95% CI 96–99) at 12 months, 94% (95% CI 93–95) at 24 months, 89% (95% CI 88–90) at 36 months, and 85% (95% CI 83–85) at 48 months (S1 Table). When comparing women and men, crude retention rates were 99%, 95%, 91%, and 87% for women at 12, 24, 36, and 48 months, respectively, compared to 98%, 93%, 86%, and 80% for men over the same time periods.

Furthermore, patients with a CD4 count of 100–199 cells/μL had significantly higher retention in care compared to those with CD4 counts of 0–99 cells/μL (Log-rank, X^2^ = 5.77, p = 0.0163). Similarly, those who initiated ART within 7 days had higher retention compared to those who initiated ART after 8 days or more (Log-rank, X^2^ = 263.92, p < 0.001). Additionally, patients without TB exhibited higher retention in care than those with TB (Fig 2).

**Fig 2:**
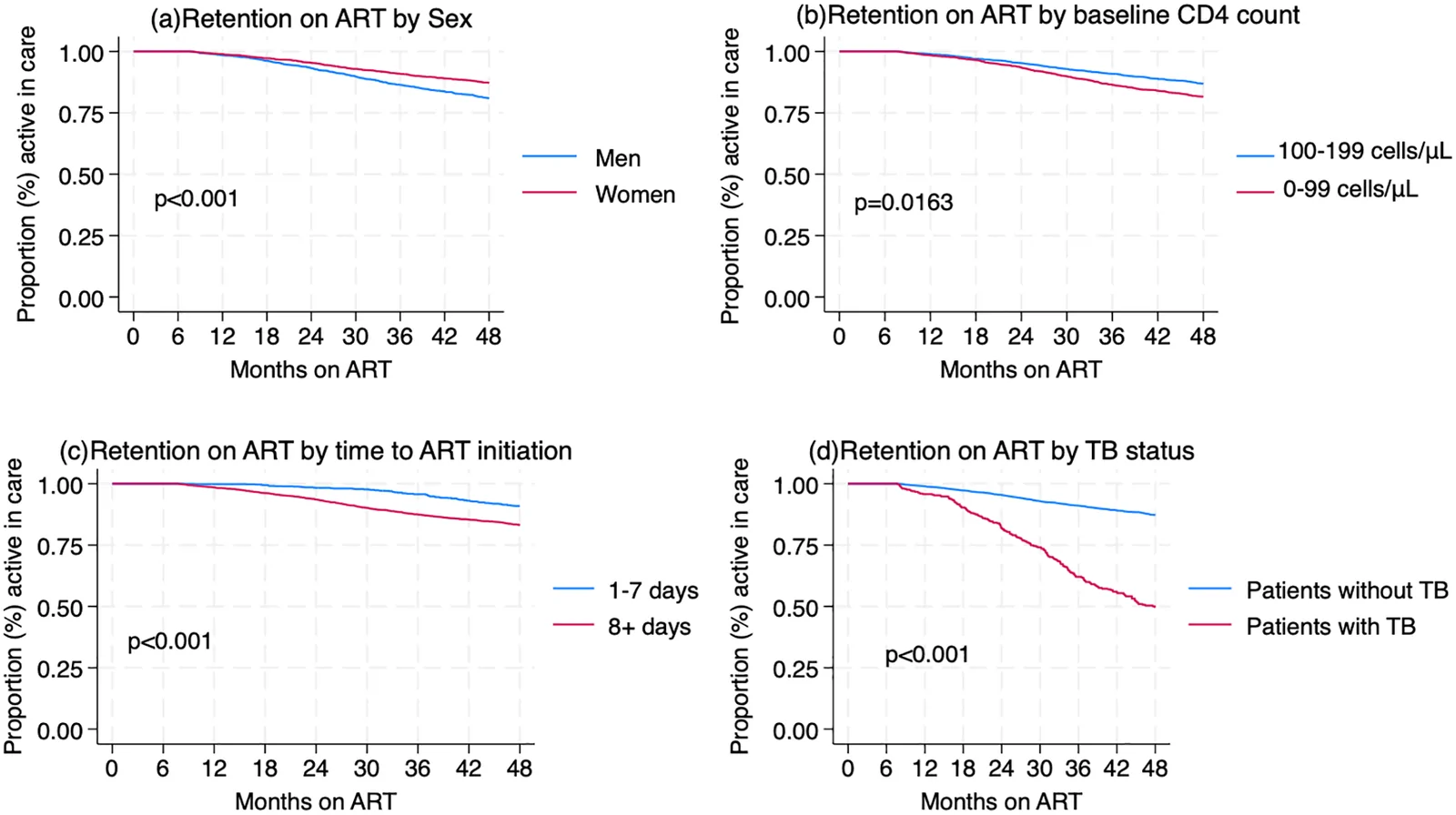
Kaplan-Meier plots of retention on antiretroviral therapy for baseline characteristics among patients with advanced HIV disease. ART, antiretroviral therapy; TB, Tuberculosis.

### 3.4. Factors associated with loss-to-follow among patients with AHD

In the multivariable competing risks regression model of time to LTFU accounting for the competing risk of death (Table 3), Men had a 35% higher subdistribution hazard of LTFU compared (adjusted sub-distribution hazard ratio [aSHR], 1.35; 95% CI 1.23–1.476) to women. Patients with CD4 of of 0–99 cells/μL had a 13% higher subdistribution hazard of LTFU (aSHR, 1.13; 95% CI 1.08–1.26) compared to those with a CD4 count of 100–199 cells/μL. Conversely, patients without TB had a significantly lower subdistribution hazard of LTFU (aSHR 0.60; 95% CI 0.39–0.94) compared to those with TB. Similarly, patients enrolled in HIV care during the UTT era had a lower subdistribution hazard of LTFU (aSHR 0.48; 95% CI 0.43–0.68) compared to those enrolled between 2008 and 2012.

**Table 3:** Factors associated with loss-to-follow-up among patients with AHD initiated on first-line ART, adjusted for death as Competing Risk.

Variables	Crude SHR (95% CI)	P-values	Adjusted SHR* (95% CI)	P-values
**Sex**				
Women	Reference		Reference	
Men	1.23 (1.13-1.34)	<0.001	1.35 (1.23-1.47)	<0.001
**Age categories**				
18-24	Reference		Reference	
25-31	0.79 (0.69-0.91)	0.001	0.71 (0.61-0.82)	<0.001
32-38	0.66 (0.57-0.77)	<0.001	0.56 (0.48-0.64)	<0.001
39-45	0.58 (0.50-0.69)	<0.001	0.47 (0.40-0.56)	<0.001
>45	0.52 (0.43-0.61)	<0.001	0.41 (0.37-0.46)	<0.001
**Baseline CD4 counts**				
0-99 cells/μL	Reference		Reference	
100-199 cells/μL	1.15 (1.05-1.25)	0.001	1.13 (1.08-1.26)	0.022
**Patients with Tuberculosis**				
No	0.63 (0.50-0.79)	<0.001	0.60 (0.39-0.94)	0.025
Yes	Reference		Reference	
**ART regimen on enrolment**				
ABC + 3TC	Reference		Reference	
AZT + 3TC	0.78 (0.38-1.55)	0.489	0.72 (0.53-0.97)	0.035
TDF + FTC/3TC + EFV	0.79 (0. 44-1.40)	0.416	0.66 (0.41-1.06)	0.091
**Time-ART Initiation**				
1-7 days	Reference		Reference	
8 + days	1.06 (0.95-1.19)	0.273	1.18 (1.06-1.32)	0.002
**Year of ART initiation**				
2008-2011	Reference		Reference	
2012-2015	1.23 (1.12-1.36)	<0.001	1.09 (0.88-1.36)	0.392
2016-2020	0.84 (0.72-0.95)	0.008	0.48 (0.43-0.68)	0.003
Duration on ART**				
Month on ART	0.96 (0.95-0.97)	<0.001	0.96 (0.95-0.97)	<0.001

ABC, abacavir; AHD, advanced HIV disease; ART, antiretroviral therapy; FTC, Emtricitabine; EFV, efavirenz; SHR, subdistribution hazard ratio; AZT, zidovudine; CI, confidence interval; TDF, tenofovir disoproxil fumarate; 3TC, Lamivudine. *Age categories were adjusted for sex, while all other variables in the adjusted analysis were constructed separately, each adjusted for both sex and age categories.* *Duration on ART was treated as continuous variable.

Furthermore, patients with AHD in the age groups 25–31 years, 32–38 years, and 39–45 years demonstrated a subdistribution hazard of LTFU compared to those aged 18–24 years, with (aSHR 0.71; 95% CI, 0.61–0.82), (aSHR 0.56; 95% CI 0.48–0.64), and (aSHR 0.47; 95% CI 0.40–0.56), respectively. Notably, for every additional month on ART, the subdistribution hazard of LTFU decreased by 4% (aSHR 0.96; 95% CI 0.95–0.97).

Finally, patients who initiated ART with a lower CD4 count of 0–99 cells/μL experienced a higher cumulative incidence of LTFU compared to with a CD4 count of 100–199 cells/μL (S1 Fig). Similarly, patients enrolled in HIV care during the UTT had a lower cumulative incidence of LTFU compared to those enrolled between 2008 and 2011. Additionally, patients with TB exhibited a higher cumulative incidence of LTFU compared to those without TB. However, there was no difference in cumulative incidence of LTFU between women and men.

### 3.5. Viral load suppression and undetectable viral load among patients with AHD and factors associated with virological suppression

Among 9,608 patients who initiated ART with AHD, VL completion was 49.3% at 6 months, 46.5% at 12 months, 43.4% at 24 months, 37.0% at 36 months, and 31.5% at 48 months (S2 Table). The overall virological suppression was 83.5% (95% CI 82.1–85.0) at 6 months, 85.4% (95% CI 84.0–86.7) at 12 months, 90.1% (95% CI 88.7–91.2) at 24 months, 92.4% (95% CI 91.0–93.5) at 36 months, and 88.3% (95% CI 87.1–98.4) 48 months of ART (Table 4), whereas undetectable VL was 24.0% (95% CI 22.7–25.2) at 6 months, 25.5% (95% CI 24.2–27.0) at 12 months, 26.4% (95% CI 25.1–27.8) at 24 months, 29.5% (95% CI 28.0–31.0) at 36 months, and 33.0% (95% CI 31.2–34.5) at 48 months of ART. Furthermore, patients who initiated ART between 2016–2020 exhibited a higher proportion of undetectable VL. VL suppression was comparable between the 2012–2015 and 2016–2020 cohorts.

**Table 4:** Viral suppression at 6, 12-, 24-, 36, and 48- months among patients with AHD, by period of ART initiation.

VL suppression (<1000 copies/μL), proportion (95% CI)				
	All N = 9,608	2008-2011 N = 4,754	2012-2015 N = 4,465	2016-2020 N = 4,170
6 months	83.5% (82.1-85.0)	74.4% (72.0-77.0)	82.0% (79.0-83.8)	85.3% (83.3-87.1)
12 months	85.4% (84.0-86.7)	73.0% (70.5-75.2)	84.4% (82.3-86.3)	86.4% (84.3-88.2)
24 months	90.1% (88.7-91.2)	72.0% (70.0-74.6)	90.0% (88.2-91.6)	90.1% (88.0-92.0)
36 months	92.4% (91.0-93.5)	72.1% (69.6-74.5)	93.0% (91.1-94.2)	91.4% (88.8-93.5)
48 months	88.3% (87.1-98.4)	82.1% (80.0-84.7)	92.1% (87.3-93.3)	90.7% (87.3-93.3)
Undetectable VL (<50 copies/μL), proportion (95% CI)				
6 months	24.0% (22.7-25.2)	10.4% (8.9-12.3)	19.5% (18.0-21.2)	43.4% (41.0-46.1)
12 months	25.5% (24.2-27.0)	9.2% (7.8-9.2)	22.3% (20.4-24.2)	49.4% (46.6-52.2)
24 months	26.4% (25.1-27.8)	9.0% (7.6-10.6)	25.1% (23.2-27.2)	57.0% (53.5-60.0)
36 months	29.5% (28.0-31.0)	10.0% (8.4-11.7)	30.6% (28.5-33.0)	72.0% (68.2-75.6)
48 months	33.0% (31.2-34.5)	14.6% (12.7-16.7)	36.3% (33.8-38.7)	78.0% (73.5-82.0)

In the multivariate binomial regression analysis (Table 5), women were more likely to achieve virological suppression below 1000 copies/μL compared to men (Risk ratio [RR], 1.6; 95% CI 1.1–1.6). Additionally, patients who initiated ART with a higher CD4 count of 100–199 cells/μL were more likely to achieve virological suppression (RR 1.15; 95% CI 1.10–1.22) compared-those with a CD4 count of 0–99 cells/μL.

**Table 5:** Factors associated with viral suppression (<1000 copies/μL) at any time point among patients with AHD on ART adjusted for clustering at facility level.

Variables	Viral load (N = 9,608)		Crude RR (95% CI)	Adjusted RR (95% CI)
	Unsuppressed	Suppressed		
**Sex, n (%)**				
Women	1,860 (33.9)	3,621 (66.1)	1.31 (1.08-1.25)	1.18 (1.11-1.25)
Men	1,786 (43.3)	2,341 (56.7)	Reference	Reference
**Age categories n (%)**				
18-24	409 (45.1)	498 (54.9)	Reference	Reference
25-31	1,089 (41.2)	1,570 (58.8)	1.07 (1.08-1.25)	1.11 (1.04-1.18)
32-38	987 (38.9)	1,689 (63.1)	1.15 (1.05-1.25)	1.22 (1.15-1.31)
39-45	595 (34.8)	1,115 (65.2)	1.19 (1.10-1.28)	1.26(1.20-1.33)
>45	557 (33.8)	1,090 (66.2)	1.20 (1.10-1.31)	1.28 (1.20-1.37)
**Baseline CD4 counts, n (%)**				
0-99 cells/μL	1,862 (44.0)	2,376 (56.0)	Reference	Reference
100-199 cells/μL	1,784 (33.2)	3,586 (66.8)	1.19 (1.09-1.30)	1.17 (1.10-1.27)
**Tuberculosis, n (%)**				
No	3,394 (37.8)	5,584 (62.2)	Reference	Reference
Yes	252 (40.0)	378 (60.0)	0.96 (0.87-1.06)	1.01 (0.93-1.09)
**ART regimen on enrolment, n (%)**				
ABC + 3TC	30 (51.7)	28 (48.3)	Reference	Reference
AZT + 3TC	57 (40.7)	83 (59.3)	1.22 (0.99-1.51)	1.24 (1.02-1.50)
TDF + FTC/3TC + EFV	2,607 (35.5)	4,735 (64.5)	1.33 (1.12-1.58)	1.41 (1.20-1.65)
**Time-ART Initiation, n (%)**				
1-7 days	397 (24.5)	1,227 (75.5)	1.27 (1.11-1.46)	1.28 (1.12-1.45)
8 days or more	3,249 (40.7)	4,735 (59.3)	Reference	Reference
**Year of ART initiation, n (%)**				
2008-2011	1,467 (43.9)	1,953 (57.1)	Reference	Reference
2012-2015	1,271 (33.8)	2,490 (66.2)	1.16 (1.04-1.28)	1.19 (1.08-1.32)
2016-2020	908 (37.4)	1,519 (62.6)	1.10 (0.97-1.23)	1.13 (1.01-1.28)

Data are n (%). Percentages rows were calculated with the total in each category as the denominator. All other percentages used rows as denominators. ABC, abacavir; AHD, advanced HIV disease; ART, antiretroviral therapy; FTC, Emtricitabine; EFV, efavirenz;SHR, sub-distribution hazard ratio; AZT, zidovudine; CI, confidence interval; TDF, tenofovir disoproxil fumarate; 3TC, Lamivudine; RR, risk ratio

Early initiation of ART within seven days was associated with a higher risk of virological suppression (RR 1.30; 95% CI, 1.04–1.38) compared to initiation after eight or more days. Patients receiving a TDF + FTC/3TC+EFV-based regimen demonstrated a significantly higher virological suppression (RR 1.38; 95% CI, 1.19–1.60) than those on an ABC + 3TC-based regimen. Furthermore, patients enrolled in HIV care between 2012 and 2015 exhibited a slightly higher virological suppression (RR 1.08; 95% CI, 1.02–1.15) compared to those enrolled between 2008 and 2012. Notably, as age increased, the risk of achieving virological suppression below 1,000 copies/μL also increased. However, TB at ART initiation (RR 0.96; 95% CI, 0.89–1.05) and enrolment in HIV care during the UTT era (RR 1.06; 95% CI, 0.98–1.15) were not significantly associated with virological suppression below 1,000 copies/μL.

## 4. Discussion

In this analysis of routine clinical data from HIV service delivery in 10 public primary care clinics in rural KwaZulu-Natal, we characterized AHD and its outcomes in a cohort of PLHIV who initiated ART between 2008 and 2020. This study addresses a critical gap in the literature by contributing longitudinal evidence on clinical outcomes among patients with AHD, a population for whom data remain limited, particularly in high-HIV prevalence rural settings where programmatic challenges may uniquely impact treatment trajectories. After accounting for the competing risk of death, we found a substantially higher proportion of patients initiating ART were retained in care, especially among those who started treatment during the UTT era. This suggests that improvements in retention observed are not solely due to lower mortality but reflect genuine programmatic gains under UTT implementation [16,36]. Higher LTFU was associated with being male and having a lower CD4 count. Furthermore, among patients with available viral load data, viral suppression rates were consistently high, exceeding 83% at various time points. However, among those who achieved virological suppression, the proportion of patients with an undetectable viral load remained low across most treatment eras—except among individuals who initiated ART during the UTT era.

The crude retention remained consistently high throughout the study period, with 98% retention at 12 months, 89% at 36 months and 85% at 48 months. Despite these encouraging figures, retention at later time points remains below the UNAIDS target of 95%, indicating ongoing challenges in sustaining long-term engagement in care. However, these results are in keeping with findings from a systematic review and meta-analysis, which reported retention rates for patients with AHD ranging from 23% to 97% at 12 months in Sub-Saharan Africa [37]. Numerous studies have highlighted that disengagement from HIV care is a widespread issue and significantly contributes to the progression of AHD globally [38]. The reasons for disengagement are multifactorial, encompassing structural and clinical challenges—such as long distances to health facilities and extended waiting times as well as psychosocial barriers, including stigma and discrimination [39].

Our study presents retention data beyond 12 months from ART initiation. The relatively higher retention observed in our study is likely attributable to the implementation of a series of community-based interventions at 10 public health facilities where the study was conducted [19]. This included intervention to improve linkage to care, the scale-up of differentiated models of care, enhanced adherence counselling, and improved quality of care delivered through clinical mentoring for both patients with and without AHD, implemented between 2013 and 2018 [40]. Additionally, we found that the risk of LTFU was higher among men compared to women, adding to the existing body of evidence that highlights the persistent failure of HIV services to adequately engage men in care. Our analysis also demonstrated that patients with AHD who had TB at the time of ART initiation were at a higher risk of LTFU than those without TB. This finding is consistent with previous studies [41,42] showing that individuals with AHD and TB experience poorer retention in care, largely due to the increased treatment burden associated with concurrent HIV and TB therapy, debilitating medication side effects, psychosocial barriers such as stigma and fear of disclosure, food insecurity, and the financial costs of frequent clinic visits, all of which can hinder sustained engagement in HIV care.

In our study, we observed consistently high rates of viral suppression. The overall virological suppression rates were 85.4%, 90.0%, 92.4%, and 88.3% at 12, 24, 36, and 48 months, respectively. These findings are comparable to those reported in a population-based survey conducted in the same setting in 2018, which indicated that 94% of PLHIV on ART were virally suppressed. Given that our study focused exclusively on patients with AHD, these outcomes represent a favorable virologic outcome within this high-risk subgroup. However, the viral suppression rates reported in our study are slightly lower than those observed in Botswana, where suppression rates ranged from 87% to 92% [43,44]. Nevertheless, our results are notably higher than those reported in other rural Rwandan and Nigerian districts, where suppression rates ranged between 63% and 75% [41,45,46]. Despite the overall impressive virologic outcomes across population groups and clinical characteristics, men consistently had the lowest rates of viral suppression, consistent with findings reported in other settings. [41,44].

Additionally, among those who achieved virological suppression, the proportion of patients with an undetectable viral load remained relatively low overall- with 25.5%, 26.4%, 29.5%, and 33.0% at 12, 24, 36, and 48 months, respectively. Although higher proportions were observed among individuals who initiated ART during the UTT era compared to those in earlier periods, with 49.4%, 57.0%, 72.0% and 78.0% at at 12, 24, 36, and 48 months, respectively. Given the evidence that PLHIV who are virally suppressed do not sexually transmit HIV [47], targeted strategies are needed to improve viral suppressiona and to further reduce HIV transmission [48]. These strategies, including enhanced adherence counselling, cognitive behavioural therapy, and psychosocial support should be targeted toward improving both overall viral suppression and the proportion of individuals achieving an undetectable viral load, with particular emphasis on reaching men [49].

The strengths of this study include its contribution to the growing body of evidence evaluating long-term treatment outcomes among patients with AHD under routine programmatic conditions, particularly in high HIV prevalence, low-income rural settings. A key methodological strength of this study is the use of routine programmatic data from a large patient cohort receiving services in a rural sub-district, which enhances the relevance of our findings to real-world programmatic contexts. The sample size also strengthens the external validity of our results. Notably, the inclusion of substantial HIV viral load data added significant value to our analysis. Viral load testing remains limited in many parts of sub-Saharan Africa, especially in rural clinics where CD4 count monitoring and clinical criteria are often the primary tools for patient assessment [50].

Although informative, our study has several limitations that may affect both the internal and external validity of the findings. First, the retrospective nature of the study design limited our ability to collect certain variables of interest, such as the provision of other prophylactic treatments, such as cotrimoxazole prophylaxis, fluconazole pre-emptive therapy and the presence of opportunistic infections beyond TB. Second, the programmatic nature of the dataset poses challenges, as the data were not originally collected for research purposes and may be subject to reporting inconsistencies or missing information. Finally, the generalizability of our findings to other rural settings in South Africa is limited. The study setting implemented a series of community-based interventions aimed at increasing HIV testing awareness, improving ART linkage to care, enhancing retention and adherence, and scale up differentiated models of care. Additionally, enhanced adherence counselling and strengthened quality of care—delivered through clinical mentoring for both patients with and without AHD were introduced [40]. These These programmatic interventions likely contributed to the positive outcomes observed.

## 5. Conclusion

This study provides important insights into the long-term programmatic outcomes of patients with AHD in a high HIV-prevalence, rural South African setting. Despite the challenges inherent in resource-limited settings, our findings demonstrate encouraging levels of retention and virological suppression among patients with AHD who initiated ART over 12 years. Notably, improved outcomes were observed among those initiating ART during the UTT, underscoring the impact of evolving treatment policies. However, the persistently low rates of undetectable viral load highlight ongoing gaps in care that must be addressed to prevent HIV transmission. These results reinforce the critical importance of integrating the WHO-recommended AHD care package into routine HIV programs and tailoring interventions to support early ART initiation and sustained engagement in care. Targeted strategies, especially for men and those with low CD4 counts, are essential to further enhance treatment outcomes and progress towards epidemic control.

## Supporting information

S1 Table Kaplan–Meier estimates of retention according to selected variables (percentages and 95% confidence intervals). (DOCX)

S2 Table Viral load testing uptake at 6, 12, 24, 36, and 48 months among patients with advanced HIV disease (AHD), by period of antiretroviral therapy (ART) initiation. (DOCX)

S1 Fig Cumulative incidence of loss to follow-up among patients with advanced HIV disease (AHD) who initiated antiretroviral therapy (ART), adjusted for death as a competing risk. TB, tuberculosis.
(PNG)
