# Supplementary material for: Evaluating long-term programmatic outcomes of patients with advanced HIV disease: Insights from 12 years of antiretroviral therapy scale-up in rural KwaZulu-Natal
Source: PLoS One. 2026 Oct 27;21(10):e0360657. doi: 10.1371/journal.pone.0360657 (PMC13712600; doi:10.1371/journal.pone.0360657)
Supplement: Supplementary file 1 [file pone.0360657.s001.docx]

S1 Table. Kaplan–Meier estimates of retention according to selected variables (percentage and 95% confidence interval).

|  | 12 months | 24 months | 36 months | 48 months |
| --- | --- | --- | --- | --- |
| Entire Cohort | 98 (96-99) | 94 (94-95) | 89 (88-90) | 85 (83-85) |
| Sex | | | | |
| Men | 98 (97-98) | 93 (92-94) | 86 (85-87) | 80 (79-82) |
| Women | 99 (98-99) | 95 (94-96) | 91 (90-92) | 87 (86-88) |
| Age at HIV care enrolment (years) | | | | |
| 18-24 | 99 (97-99) | 93 (91-95) | 90 (87-92) | 83 (80-87) |
| 25-31 | 98 (98-99) | 95 (94-96) | 90 (88-91) | 85 (83-87) |
| 32-38 | 98 (97-98) | 93 (92-94) | 87 (85-88) | 87 (85-88) |
| 39-45 | 99 (98-99) | 94 (92-95) | 88 (86-90) | 84 (82-86) |
| >45 | 98 (97-99) | 95 (94-96) | 91 (89-92) | 86 (84-88) |
| Baseline CD4 counts | | | | |
| 0-99 cells/μL | 98 (98-99) | 93 (92-94) | 86 (85-88) | 81 (80-83) |
| 100-199 cells/μL | 98 (98-99) | 95 (94-96) | 91 (90-92) | 87 (85-88) |
| Tuberculosis treatment | | | | |
| No | 99 (98-99) | 95 (95-96) | 91 (90-92) | 87 (86-88) |
| Yes | 95 (93-97) | 81 (78-85) | 62 (57-67) | 50 (44-55) |
| Time on ART |  |  |  |  |
| 1-7 days | 99 (99-100) | 98 (96-99) | 95 (94-97) | 90 (89-92) |
| 8 days and more | 98 (97-99) | 93 (93-94) | 87 (86-88) | 83 (82-84) |
