# Supplementary material for: Evaluating long-term programmatic outcomes of patients with advanced HIV disease: Insights from 12 years of antiretroviral therapy scale-up in rural KwaZulu-Natal
Source: PLoS One. 2026 Oct 27;21(10):e0360657. doi: 10.1371/journal.pone.0360657 (PMC13712600; doi:10.1371/journal.pone.0360657)
Supplement: Supplementary file 2 [file pone.0360657.s002.docx]

S2 Table. Viral load testing uptake at 6-, 12-, 24-, 36-, and 48- months among patients with AHD, by period of ART initiation

| Months on ART | All  N=9,608 | 2008-2011  N=3,420 | 2012-2015  N=3,761 | 2016-2020  N=2,427 |
| --- | --- | --- | --- | --- |
| 6 months | 4,742 (49.3%) | 1,211 (35.4%) | 2,196 (58.4%) | 1,335 (55.0%) |
| 12 months | 4,465 (46.7%) | 1,347 (40.1%) | 1,898 (50.5%) | 1,193 (49.1%) |
| 24 months | 4,170 (43.4%) | 1,436 (42.0%) | 1,833 (49.0%) | 901 (37.1%) |
| 36 months | 3,542 (37.0%) | 1,322 (38.6%) | 1658 (44.1%) | 562 (23.1%) |
| 48 months | 3,030 (31.5%) | 1,190 (35.0%) | 1,471 (39.1%) | 369 (25.2%) |
