# Supplementary material for: Evaluating long-term programmatic outcomes of patients with advanced HIV disease: Insights from 12 years of antiretroviral therapy scale-up in rural KwaZulu-Natal
Source: PLoS One. 2026 Oct 27;21(10):e0360657. doi: 10.1371/journal.pone.0360657 (PMC13712600; doi:10.1371/journal.pone.0360657)
Supplement: Supplementary file 3 [file pone.0360657.s004.docx]

Title: Evaluating Long-Term Outcomes of Patients with Advanced HIV Disease: Insights from 12 Years of Antiretroviral Therapy Scale-Up in Rural KwaZulu-Natal

Authors: Marcel K. Kitenge1,2, Geoffrey Fatti 1 , Chinmay Laxmeshwar3 , Patrick D. M. C. Katoto1,4, Peter S. Nyasulu1,5

Overall impression: This is a nicely written analysis by Kitenge et al. – a retrospective cohort study looking at over 12 years of data from rural South Africa. The authors endeavor to evaluate the impact of ART on people with advanced HIV disease over a decade and more when HIV clinical care (including the robustness of the regimen) and differentiated service delivery models were increasing including options for people with AHD. I would have like to see a more nuanced description of the cohort beyond sex and age, as the inclusion of key populations or people who migrate may have affected some of the outcomes. However, as this is a retrospective study, I understand that these details may not be collected, and thus this point could be mentioned in the limitations. The strength of the study is the length of follow up and a large population to look at, with a focus on rural HIV care, which is often overlooked. I was delighted to see the authors discussed some of the differentiated service delivery model in the conclusion, but I would have liked to hear more about the DSDM coverage and which model was most useful for a rural community. It also would be interesting to mention if PrEP was introduced during this time period as well.

Major comments:

- Line 238 reads, “Additionally, patients receiving TB treatment exhibited **a lower cumulative incidence of LTFU** compared to those not receiving TB treatment.” However, when I look at your table S1 Table 1 I think you are referring to a lower incidence of retention, not LTFU?
- ART regimens: Table 1 and line 194 and 195; I have not seen studies refer to the NRTI as the primary driver of a regimen, usually it is a regimen that is based on efavirenz or more recently dolutegravir. Usually if it is a co-formulated option, it would have been TDF/FTC/EFV or more recently TDF/FTC/DTG. An experienced reader would understand that the official regimens would have changed over the 12 years of the study, but the authors should try to clarify this point and if most of the ART was efavirenz based over the 12 years or if dolutegravir was introduced. If not would just mention when this was introduced to the national program.
- 1/5 of the people have TB but we don’t know too much about this group except that they did poorly. Do the authors have additional information on this group? Any data on DR TB? Do we know what % of the 6.7% who died, died with or of TB? It may be interesting for the authors to follow up on these deaths through chart review or other strategies. Might also comment on if TB LAM was used in the national program during this time and if it has been introduced as part of the WHO package along with CrAG.
- It was good to see that the people receiving test and treat services did well overall. Could the authors comment on whether the concept of ‘starter pack’ model was used?
- Could the authors comment on any impact on IRIS on retention – if not, that could go into the limitation.
- Viral load completion could also be access to VL services – did this increase in terms of access during the study period? Might mention this.

Minor comments

- Line 308 “Nevertheless” is repeated
- Line 335 cotrimoxazole is misspelled
- Line 343 “These” repeated
