# Supplementary material for: Evaluating long-term programmatic outcomes of patients with advanced HIV disease: Insights from 12 years of antiretroviral therapy scale-up in rural KwaZulu-Natal
Source: PLoS One. 2026 Oct 27;21(10):e0360657. doi: 10.1371/journal.pone.0360657 (PMC13712600; doi:10.1371/journal.pone.0360657)
Supplement: Supplementary file 4 [file pone.0360657.s005.docx]

21 March 2026

Dear reviewers,

Re: Responses to reviewers’ comments

Please find below a point-by-point response to the issues raised by the reviewers. The changes made to the manuscript are highlighted in track changes in the revised version.

Original Title: Evaluating Long-Term Outcomes of Patients with Advanced HIV Disease: Insights from 12 Years of Antiretroviral Therapy Scale-Up in Rural KwaZulu-Natal.

**Overall impression**

This is a nicely written analysis by Kitenge et al. – a retrospective cohort study looking at over 12 years of data from rural South Africa. The authors endeavor to evaluate the impact of ART on people with advanced HIV disease over a decade and more when HIV clinical care (including the robustness of the regimen) and differentiated service delivery models were increasing including options for people with AHD. I would have like to see a more nuanced description of the cohort beyond sex and age, as the inclusion of key populations or people who migrate may have affected some of the outcomes. However, as this is a retrospective study, I understand that these details may not be collected, and thus this point could be mentioned in the limitations. The strength of the study is the length of follow up and a large population to look at, with a focus on rural HIV care, which is often overlooked. I was delighted to see the authors discussed some of the differentiated service delivery model in the conclusion, but I would have liked to hear more about the DSDM coverage, and which model was most useful for a rural community. It also would be interesting to mention if PrEP was introduced during this time period as well.

**Comment 1**

Line 238 reads, “Additionally, patients receiving TB treatment exhibited a lower cumulative incidence of LTFU compared to those not receiving TB treatment.” However, when I look at your table S1 Table 1 I think you are referring to a lower incidence of retention, not LTFU?

**Response**

Thank you for this important comment. Table S1 presents retention proportions at specific time points, whereas the statement in the manuscript refers to the cumulative incidence of LTFU derived from time-to-event analysis, as shown in Appendix Figure 1 (panel d).

While retention and LTFU are related outcomes, they are not identical measures. Retention reflects the proportion of patients remaining in care at a fixed time point, whereas cumulative incidence of LTFU accounts for the timing of events and censoring over follow-up.

In our cumulative incidence analysis, patients receiving TB treatment had a lower observed cumulative incidence of LTFU compared to those not receiving TB treatment.

**Comment 2**

ART regimens: Table 1 and line 194 and 195; I have not seen studies refer to the NRTI as the primary driver of a regimen, usually it is a regimen that is based on efavirenz or more recently dolutegravir. Usually if it is a co-formulated option, it would have been TDF/FTC/EFV or more recently TDF/FTC/DTG. An experienced reader would understand that the official regimens would have changed over the 12 years of the study, but the authors should try to clarify this point and if most of the ART was efavirenz based over the 12 years or if dolutegravir was introduced. If not would just mention when this was introduced to the national program.

**Response**

Thank you for this important comment. We have made changes accordingly as shown in line 195 and Table 1.

**Comment 3**

1/5 of the people have TB but we don’t know too much about this group except that they did poorly. Do the authors have additional information on this group? Any data on DR TB? Do we know what % of the 6.7% who died, died with or of TB? It may be interesting for the authors to follow up on these deaths through chart review or other strategies. Might also comment on if TB LAM was used in the national program during this time and if it has been introduced as part of the WHO package along with CrAG.

**Response**

We appreciate the reviewer’s observation. We have clarified the number of participants who had TB in the Results section (Lines 199 and 200), which now reads as follows “*Of the 9,608 patients included in the study, over the observation period, the majority, 6,551 (68.2%), remained in care at 48 months, while 2,428 (25.2%) were LTFU and 639 (6.7%) died. Among those who died, 32 (5.0%) had TB.”.*

Additionally, we appreciate the reviewer’s comment regarding the methods of diagnosis. Unfortunately, the data extracted by the District Health Information Manager did not include the TB module, which captures detailed information on diagnostic methods. Furthermore, the study period corresponds to the early phase of implementation of the WHO diagnostic package in public health facilities. During this period, diagnostic indicators such as CrAg testing and TB TB-LAM were not routinely captured in TIER.Net.

Furthemore, patients diagnosed with drug-resistant TB—including rifampicin-resistant TB (RR-TB), multidrug-resistant TB (MDR-TB), and other forms of drug-resistant TB —are registered in a separate electronic database (EDRWeb), rather than in TIER.Net. We were unable to triangulate data across these different databases, as ethical approval for this study was granted solely for the use of TIER.Net data.

**Comment 4**

It was good to see that the people receiving test and treat services did well overall. Could the authors comment on whether the concept of ‘starter pack’ model was used?

**Response**

We thank the reviewer for this insightful comment. The “starter pack” model, as described in some settings (e.g., Malawi), is not a formally adopted or nationally recognized model within the South African HIV programme. In the South African context, ART initiation under the Universal Test and Treat (UTT) strategy follows standardized national treatment guidelines, which include provision of an initial supply of first-line antiretroviral therapy at the time of initiation.

While facilities may provide an initial pre-packaged supply of ART at treatment initiation, this is implemented as part of routine guideline-based care rather than as a distinct “starter pack” model. Therefore, the outcomes observed in our study reflect implementation of the national UTT policy and standardized ART initiation protocols, rather than a specific starter pack approach

**Comment 5**

Could the authors comment on any impact on IRIS on retention – if not, that could go into the limitation.

**Response**

We thank the reviewer for this insightful comment. We acknowledge lack of data on the IRIS as one of the limitations of our study as shown in line 346 and 347 and this reads as follow “Additionally, we were unable to evaluate the impact of immune reconstitution inflammatory syndrome (IRIS) on mortality outcomes due to data limitations.”

**Comment 6**

Line 308 “Nevertheless” is repeated

**Response**

We thank the reviewer for highlighting this repetition. The duplicated word has been removed accordingly as shown in line 310.

**Comment 7**

Line 335 cotrimoxazole is misspelled

**Response**

We appreciate the reviewer for identifying this error. The spelling has been corrected, as reflected in line 337.

**Comment 8**

Line 343 “These” repeated

**Response**

We thank the reviewer for highlighting this repetition. The duplicated word has been removed accordingly as shown in line 345.
