# Supplementary material for: Evaluating long-term programmatic outcomes of patients with advanced HIV disease: Insights from 12 years of antiretroviral therapy scale-up in rural KwaZulu-Natal
Source: PLoS One. 2026 Oct 27;21(10):e0360657. doi: 10.1371/journal.pone.0360657 (PMC13712600; doi:10.1371/journal.pone.0360657)
Supplement: Supplementary file 5 [file pone.0360657.s006.docx]

27 June 2026

Dear reviewers,

Re: Responses to reviewers’ comments

Please find below a point-by-point response to the issues raised by the reviewers. The changes made to the manuscript are highlighted in track changes in the revised version.

Original Title: Evaluating Long-Term Outcomes of Patients with Advanced HIV Disease: Insights from 12 Years of Antiretroviral Therapy Scale-Up in Rural KwaZulu-Natal.

**Overall impression**

This is a nicely This publication adds a significant contribution to the body of work on advanced HIV disease in South Africa, especially for rural South Africa. It covered data from 2008 to 2020 and highlighted better retention amongst those with Advanced HIV Disease who initiated art in the universal test and treat era, including lower mortality and better viral load suppression. The study also highlighted gaps in viral load completion. General comments, please review spelling in the document, including figures and tables.

**Comment 1**

Line 184: Missing a “1) for the first time period: 2008-2011

**Response**

Thank you for this important comment. We have addressed the comment as shown 185-186 in line and this reads as follows “*Of these 3,420 (35.6%) initiated ART during the 2008-2011 period, 3,761 (39.1%) initiated ART during 2012 and 2015, and 2,427 (49.3%) initiated during the Universal Test and Treat (UTT) (2016-2020)*”

**Comment 2**

Line 184: The start year for UTT period is incorrectly labelled as 2013 instead of 2016 as noted in other parts of the manuscript including table 1.

**Response**

Thank you for this important comment. We have made changes accordingly as shown in line 186.

**Comment 3**

Lines 222-229: The numbers for the adjusted Hazard ratios and 95% presented in the manuscript differ from the numbers shown on Table 3. Please review numbers and statements to ensure alignment with Table 3.

a. For example, The author states that patients with lower CD4 had a 15% higher sub distribution of LTFU
however table three, line 564 shows that those with a higher CD4 of 100 to 199 had a 13% higher sub distribution hazard of LTFU.
b. For certain variables the author referenced the crude hazard ratio while for others, referenced the adjusted hazard ratio. I could recommend presenting the adjusted results.

**Response**

We appreciate the reviewer's careful observation. We have corrected the discrepancies between the effect estimates and 95% confidence intervals reported in the text and those presented in Table 3. The revised text now aligns with Table 3, and these changes have been made between lines 225 and 238. Additionally, to improve consistency and avoid confusion, we have presented only the adjusted effect estimates throughout the manuscript.

**Comment 4**

Lines 235-236: Finally, patients who initiated ART with a lower CD4 count of 0-99 cells/μL experienced a lower cumulative incidence of LTFU compared to with a CD4 count of 100-199 cells/μL (appendix Figure1).
a. This statement is not supported by the data in Appendix fig 1 which shows a higher cumulative incidence of LTFU for those with CD4 of 0-99 cells/μL

**Response**

We thank the reviewer for this insightful comment. We have revised the manuscript accordingly. The relevant changes have been incorporated in line 239 and now read as follows: “Finally, patients who initiated ART with a lower CD4 count of 0-99 cells/μL experienced a higher cumulative incidence of LTFU compared to with a CD4 count of 100-199 cells/μL (appendix Figure 1).”

**Comment 5**

Lines 238-239: Additionally, patients receiving TB treatment exhibited a lower cumulative incidence of LTFU compared to those not receiving TB treatment, according to Figure 2.
a. This statement is not supported by the data shown on Table 3. According to that table 3 patients not on TB treatment had a lower hazards of LTFU. Recommend double checking reference groups.

b. Please clarify the denominator for this statement? Is this among the total number of patients with AHD or among those with confirmed TB? If the denominator is among those with confirmed TB who are eligible for treatment, then your findings align with the fact that patients who are engaged in TB treatment are less likely to be LTFU.

**Response**

We thank the reviewer for this insightful comment. We have revised the manuscript accordingly. The relevant changes have been incorporated in line 242-244 and now read as follows: “*Additionally, patients with TB exhibited a higher cumulative incidence of LTFU compared to those without TB. However, there was no difference in cumulative incidence of LTFU between women and men*.”

We appreciate the reviewer's careful observation. We have corrected this throughout the manuscript. Specifically, of the 9,608 patients with advanced HIV disease (AHD), 630 (6.6%) had TB at the time of ART initiation. This has been corrected in line 190 and now reads: *"Additionally, 6.6% of patients had TB at the time of ART initiation.”.* To improve clarity and ensure consistent terminology, we have replaced references to "TB treatment" with "patients with TB" where appropriate. These revisions have been made in lines 190–191, 222–223, 229–231, 242–243, and 352–353.

**Comment 6**

Line Line 237: “Similarly, patients enrolled in HIV care during the UTT had a lower cumulative incidence of LTFU compared to those enrolled between 2008 and 2012.”
a. This should be 2008-2011 Based on the time period Specified in the methods and table one, instead of 2008-2012 per manuscript.

**Response**

We thank the reviewer for highlighting these inconsistencies. We have corrected them accordingly. The revised text has been incorporated in lines 241–243 and now reads as follows *“Similarly, patients enrolled in HIV care during the UTT had a lower cumulative incidence of LTFU compared to those enrolled between 2008 and 2011”.*

**Comment 7**

Table 4: missing column total for the Ns for the three time periods.

**Response**

We appreciate the reviewer's comment. We have included the missing total (N) column for each of the three time periods, as requested. These revisions have been incorporated in Table 4 and are reflected in lines 648–650.

**Comment 8**

Given the high prevalence of TB in PLHIV in South Africa, it is important to address in the discussion some possible reasons why those not receiving TB treatment had higher retention (lines 218-219) and a significantly lower sub-distribution hazard of LTFU than those receiving TB treatment (line 225-227).

**Response**

We thank the reviewer for this valuable suggestion. We have added a paragraph discussing the association between tuberculosis (TB) at ART initiation and retention in care. This addition has been incorporated in lines 389–395 and now reads as follows: *“Our analysis also demonstrated that patients with AHD who had TB at the time of ART initiation were at a higher risk of LTFU than those without TB. This finding is consistent with previous studies (41, 42) showing that individuals with AHD and TB experience poorer retention in care, largely due to the increased treatment burden associated with concurrent HIV and TB therapy, debilitating medication side effects, psychosocial barriers such as stigma and fear of disclosure, food insecurity, and the financial costs of frequent clinic visits, all of which can hinder sustained engagement in HIV care.”.*
