# Supplementary material for: Evaluating long-term programmatic outcomes of patients with advanced HIV disease: Insights from 12 years of antiretroviral therapy scale-up in rural KwaZulu-Natal
Source: PLoS One. 2026 Oct 27;21(10):e0360657. doi: 10.1371/journal.pone.0360657 (PMC13712600; doi:10.1371/journal.pone.0360657)
Supplement: Supplementary file 6 [file pone.0360657.s007.docx]

04 September 2026

Dear reviewers,

Re: Responses to reviewers’ comments

Please find below a point-by-point response to the issues raised by the reviewers. The changes made to the manuscript are highlighted in track changes in the revised version.

Original Title: Evaluating Long-Term Outcomes of Patients with Advanced HIV Disease: Insights from 12 Years of Antiretroviral Therapy Scale-Up in Rural KwaZulu-Natal.

**Comment 1**

The authors made updates to the TB results and noted in their response: "To improve clarity and ensure consistent terminology, we have replaced references to "TB treatment" with "patients with TB" where appropriate." Changes were made to the text, but TB treatment is still referenced on Table 3 and Fig 2 instead of TB status at ART initiation. (legend labels).

**Response**

We appreciate the reviewer for the careful observation. We have revised the manuscript accordingly. The relevant changes have been incorporated in line 562 or Table 3 and line 558 or Figure 2.

**Comment 2**

Fig panel D: retention is misspelled.

**Response**

We appreciate the reviewer one more time for the careful observation. We have revised this as shown in line 558 or Figure 2, panel D.

**Comment 3**

Line 344, Cotrimoxazole is still misspelled as "contimoxazole".

**Response**

We thank the reviewer for this insightful comment. We have revised the manuscript accordingly. The relevant change has been incorporated in line 341-342 and now read as follows: “*such as cotrimoxazole prophylaxis, fluconazole pre-emptive therapy and the presence of opportunistic infections beyond TB*”.

**Comment 3**

Reviewer 1 inquired about the ART regimen; since Table 3 and 5 show the backbone (TDF+FTC/3TC), a footnote should be added clarifying the remaining ART regimen (EFV or DTG as indicated ) since the subheading states (ART regimen) and gives the impression that patients were treated with 2 drug combinations.

**Response**

Thank you for this important observation. We agree that presenting only TDF + FTC/3TC under the ART regimen heading may give the impression that participants were receiving a two-drug combination. We have therefore revised Tables 3 and 5 to clarify that TDF + FTC/3TC represented the NRTI backbone, with EFV as the third antiretroviral agent. The complete regimen is therefore presented as TDF + FTC/3TC + EFV, which reflects the use of either FTC or 3TC together with TDF and EFV.

During the study period, dolutegravir (DTG) had not yet been widely implemented across South African public health facilities, and EFV-based regimens remained commonly used. We have therefore retained EFV as the third agent in the description of the ART regimen to accurately reflect treatment practice during the study period. These changes are shown in **table 1, table 3 and table 5**.
