# Supplementary material for: Evaluating long-term programmatic outcomes of patients with advanced HIV disease: Insights from 12 years of antiretroviral therapy scale-up in rural KwaZulu-Natal
Source: PLoS One. 2026 Oct 27;21(10):e0360657. doi: 10.1371/journal.pone.0360657 (PMC13712600; doi:10.1371/journal.pone.0360657)
Supplement: Peer review history

# Peer review: Decision Letter 0

**DOI:** 10.1371/journal.pone.0360657.r001

**Authors:** Okikiolu Badejo

22 Jan 2026

Dear Dr. Kitenge,

Thank you for submitting your manuscript to PLOS ONE. After careful consideration, we feel that it has merit but does not fully meet PLOS ONE’s publication criteria as it currently stands. Therefore, we invite you to submit a revised version of the manuscript that addresses the points raised during the review process.

Please submit your revised manuscript by Mar 08 2026 11:59PM. If you will need more time than this to complete your revisions, please reply to this message or contact the journal office at plosone@plos.org. When you're ready to submit your revision, log on to https://www.editorialmanager.com/pone/ and select the 'Submissions Needing Revision' folder to locate your manuscript file.

- A letter that responds to each point raised by the academic editor and reviewer(s). You should upload this letter as a separate file labeled 'Response to Reviewers'.
- A marked-up copy of your manuscript that highlights changes made to the original version. You should upload this as a separate file labeled 'Revised Manuscript with Track Changes'.
- An unmarked version of your revised paper without tracked changes. You should upload this as a separate file labeled 'Manuscript'.

If you would like to make changes to your financial disclosure, please include your updated statement in your cover letter. Guidelines for resubmitting your figure files are available below the reviewer comments at the end of this letter.

If applicable, we recommend that you deposit your laboratory protocols in protocols.io to enhance the reproducibility of your results. Protocols.io assigns your protocol its own identifier (DOI) so that it can be cited independently in the future. For instructions see: https://journals.plos.org/plosone/s/submission-guidelines#loc-laboratory-protocols. Additionally, PLOS ONE offers an option for publishing peer-reviewed Lab Protocol articles, which describe protocols hosted on protocols.io. Read more information on sharing protocols at https://plos.org/protocols?utm_medium=editorial-email&utm_source=authorletters&utm_campaign=protocols.

We look forward to receiving your revised manuscript.

Kind regards,

Okikiolu Badejo, M.D., Ph.D

Academic Editor

PLOS One

Journal Requirements:

When submitting your revision, we need you to address these additional requirements.

1.Please ensure that your manuscript meets PLOS ONE's style requirements, including those for file naming. The PLOS ONE style templates can be found at

https://journals.plos.org/plosone/s/file?id=wjVg/PLOSOne_formatting_sample_main_body.pdf and

https://journals.plos.org/plosone/s/file?id=ba62/PLOSOne_formatting_sample_title_authors_affiliations.pdf

2. We note that you have indicated that there are restrictions to data sharing for this study. PLOS only allows data to be available upon request if there are legal or ethical restrictions on sharing data publicly. For more information on unacceptable data access restrictions, please see http://journals.plos.org/plosone/s/data-availability#loc-unacceptable-data-access-restrictions.

Before we proceed with your manuscript, please address the following prompts:

a) If there are ethical or legal restrictions on sharing a de-identified data set, please explain them in detail (e.g., data contain potentially identifying or sensitive patient information, data are owned by a third-party organization, etc.) and who has imposed them (e.g., a Research Ethics Committee or Institutional Review Board, etc.). Please also provide contact information for a data access committee, ethics committee, or other institutional body to which data requests may be sent.

b) If there are no restrictions, please upload the minimal anonymized data set necessary to replicate your study findings to a stable, public repository and provide us with the relevant URLs, DOIs, or accession numbers. For a list of recommended repositories, please see

https://journals.plos.org/plosone/s/recommended-repositories. You also have the option of uploading the data as Supporting Information files, but we would recommend depositing data directly to a data repository if possible.

We will update your Data Availability statement on your behalf to reflect the information you provide.

3. Thank you for uploading your study's underlying data set. Unfortunately, the repository you have noted in your Data Availability statement does not qualify as an acceptable data repository according to PLOS's standards.

At this time, please upload the minimal data set necessary to replicate your study's findings to a stable, public repository (such as figshare or Dryad) and provide us with the relevant URLs, DOIs, or accession numbers that may be used to access these data. For a list of recommended repositories and additional information on PLOS standards for data deposition, please see https://journals.plos.org/plosone/s/recommended-repositories.

4. If the reviewer comments include a recommendation to cite specific previously published works, please review and evaluate these publications to determine whether they are relevant and should be cited. There is no requirement to cite these works unless the editor has indicated otherwise.

[Note: HTML markup is below. Please do not edit.]

Reviewer's Responses to Questions

**Comments to the Author**

1. Is the manuscript technically sound, and do the data support the conclusions?

Reviewer #1: Yes

Reviewer #2: Yes

**********

2. Has the statistical analysis been performed appropriately and rigorously?

Reviewer #1: I Don't Know

Reviewer #2: Yes

**********

3. Have the authors made all data underlying the findings in their manuscript fully available?

The PLOS Data policy

Reviewer #1: Yes

Reviewer #2: Yes

**********

4. Is the manuscript presented in an intelligible fashion and written in standard English?

Reviewer #1: Yes

Reviewer #2: Yes

**********

Reviewer #1: Please see the attached uploaded document for my full comments. This is a nicely written analysis by Kitenge et al. – a retrospective cohort study looking at over 12 years of data from rural South Africa. The authors endeavor to evaluate the impact of ART on people with advanced HIV disease over a decade and more when HIV clinical care (including the robustness of the regimen) and differentiated service delivery models were increasing including options for people with AHD. I would have like to see a more nuanced description of the cohort beyond sex and age, as the inclusion of key populations or people who migrate may have affected some of the outcomes. However, as this is a retrospective study, I understand that these details may not be collected, and thus this point could be mentioned in the limitations. The strength of the study is the length of follow up and a large population to look at, with a focus on rural HIV care, which is often overlooked. I was delighted to see the authors discussed some of the differentiated service delivery model in the conclusion, but I would have liked to hear more about the DSDM coverage and which model was most useful for a rural community. It also would be interesting to mention if PrEP was introduced during this time period as well. See uploaded word document for specific suggestions.

Reviewer #2: This publication adds a significant contribution to the body of work on advanced HIV disease in South Africa especially for rural South Africa. It covered data from 2008 to 2020 and highlighted better retention amongst those with Advanced HIV Disease who initiated art in the universal test and treat era including lower mortality and better viral load suppression. The study also highlighted gaps in viral load completion.

General comments: please review spelling in document including figures and tables.

Results

1. Line 184: Missing a “1) for the first time period: 2008-2011

2. Line 184: The start year for UTT period is incorrectly labelled as 2013 instead of 2016 as noted in Other parts of the manuscript including table 1.

3. Lines 222-229: The numbers for the adjusted Hazard ratios and 95% presented in the manuscript differ from the numbers shown on Table 3. Please review numbers and statements to ensure alignment with Table 3.

a. For example, The author states that patients with lower CD4 had a 15% higher sub distribution of LTFU

however table three, line 564 shows that those with a higher CD4 of 100 to 199 had a 13% higher sub distribution hazard of LTFU.

b. For certain variables the author referenced the crude hazard ratio while for others, referenced the adjusted hazard ratio. I could recommend presenting the adjusted results.

4. Lines 235-236: Finally, patients who initiated ART with a lower CD4 count of 0-99 cells/μL experienced a lower cumulative incidence of LTFU compared to with a CD4 count of 100-199 cells/μL (appendix Figure1).

a. This statement is not supported by the data in Appendix fig 1 which shows a higher cumulative incidence of LTFU for those with CD4 of 0-99 cells/μL

5. Lines 238-239: Additionally, patients receiving TB treatment exhibited a lower cumulative incidence of LTFU compared to those not receiving TB treatment, according to Figure 2.

a. This statement is not supported by the data shown on Table 3. According to that table 3 patients not on TB treatment had a lower hazards of LTFU. Recommend double checking reference groups.

b. Please clarify the denominator for this statement? Is this among the total number of patients with AHD or among those with confirmed TB? If the denominator is among those with confirmed TB who are eligible for treatment, then your findings align with the fact that patients who are engaged in TB treatment are less likely to be LTFU.

6. Line 237: “Similarly, patients enrolled in HIV care during the UTT had a lower cumulative incidence of LTFU compared to those enrolled between 2008 and 2012.”

a. This should be 2008-2011 Based on the time period Specified in the methods and table one, instead of 2008-2012 per manuscript.

7. Table 4: missing column total for the Ns for the three time periods.

Discussion

1. Given the high prevalence of TB in PLHIV in South Africa, it is important to address in the discussion some possible reasons why those not receiving TB treatment had higher retention (lines 218-219) and a significantly lower sub distribution hazard of LTFU than those receiving TB treatment (line 225-227).

**********

what does this mean?). If published, this will include your full peer review and any attached files.

If you choose “no”, your identity will remain anonymous but your review may still be made public.

**Do you want your identity to be public for this peer review?**  For information about this choice, including consent withdrawal, please see our Privacy Policy

Reviewer #1: No

Reviewer #2: No

**********

[NOTE: If reviewer comments were submitted as an attachment file, they will be attached to this email and accessible via the submission site. Please log into your account, locate the manuscript record, and check for the action link "View Attachments". If this link does not appear, there are no attachment files.]

To ensure your figures meet our technical requirements, please review our figure guidelines: https://journals.plos.org/plosone/s/figures

You may also use PLOS’s free figure tool, NAAS, to help you prepare publication quality figures: https://journals.plos.org/plosone/s/figures#loc-tools-for-figure-preparation.

NAAS will assess whether your figures meet our technical requirements by comparing each figure against our figure specifications.

Attachment Submitted filename: Reviewer Response.docx

# Author comment: Author response to Decision Letter 1

**DOI:** 10.1371/journal.pone.0360657.r002

28 Mar 2026

21 March 2026

Dear reviewers,

Re: Responses to reviewers’ comments

Please find below a point-by-point response to the issues raised by the reviewers. The changes made to the manuscript are highlighted in track changes in the revised version.

Original Title: Evaluating Long-Term Outcomes of Patients with Advanced HIV Disease: Insights from 12 Years of Antiretroviral Therapy Scale-Up in Rural KwaZulu-Natal.

Overall impression

This is a nicely written analysis by Kitenge et al. – a retrospective cohort study looking at over 12 years of data from rural South Africa. The authors endeavor to evaluate the impact of ART on people with advanced HIV disease over a decade and more when HIV clinical care (including the robustness of the regimen) and differentiated service delivery models were increasing including options for people with AHD. I would have like to see a more nuanced description of the cohort beyond sex and age, as the inclusion of key populations or people who migrate may have affected some of the outcomes. However, as this is a retrospective study, I understand that these details may not be collected, and thus this point could be mentioned in the limitations. The strength of the study is the length of follow up and a large population to look at, with a focus on rural HIV care, which is often overlooked. I was delighted to see the authors discussed some of the differentiated service delivery model in the conclusion, but I would have liked to hear more about the DSDM coverage, and which model was most useful for a rural community. It also would be interesting to mention if PrEP was introduced during this time period as well.

Comment 1

Line 238 reads, “Additionally, patients receiving TB treatment exhibited a lower cumulative incidence of LTFU compared to those not receiving TB treatment.” However, when I look at your table S1 Table 1 I think you are referring to a lower incidence of retention, not LTFU?

Response

Thank you for this important comment. Table S1 presents retention proportions at specific time points, whereas the statement in the manuscript refers to the cumulative incidence of LTFU derived from time-to-event analysis, as shown in Appendix Figure 1 (panel d).

While retention and LTFU are related outcomes, they are not identical measures. Retention reflects the proportion of patients remaining in care at a fixed time point, whereas cumulative incidence of LTFU accounts for the timing of events and censoring over follow-up.

In our cumulative incidence analysis, patients receiving TB treatment had a lower observed cumulative incidence of LTFU compared to those not receiving TB treatment.

Comment 2

ART regimens: Table 1 and line 194 and 195; I have not seen studies refer to the NRTI as the primary driver of a regimen, usually it is a regimen that is based on efavirenz or more recently dolutegravir. Usually if it is a co-formulated option, it would have been TDF/FTC/EFV or more recently TDF/FTC/DTG. An experienced reader would understand that the official regimens would have changed over the 12 years of the study, but the authors should try to clarify this point and if most of the ART was efavirenz based over the 12 years or if dolutegravir was introduced. If not would just mention when this was introduced to the national program.

Response

Thank you for this important comment. We have made changes accordingly as shown in line 195 and Table 1.

Comment 3

1/5 of the people have TB but we don’t know too much about this group except that they did poorly. Do the authors have additional information on this group? Any data on DR TB? Do we know what % of the 6.7% who died, died with or of TB? It may be interesting for the authors to follow up on these deaths through chart review or other strategies. Might also comment on if TB LAM was used in the national program during this time and if it has been introduced as part of the WHO package along with CrAG.

Response

We appreciate the reviewer’s observation. We have clarified the number of participants who had TB in the Results section (Lines 199 and 200), which now reads as follows “Of the 9,608 patients included in the study, over the observation period, the majority, 6,551 (68.2%), remained in care at 48 months, while 2,428 (25.2%) were LTFU and 639 (6.7%) died. Among those who died, 32 (5.0%) had TB.”.

Additionally, we appreciate the reviewer’s comment regarding the methods of diagnosis. Unfortunately, the data extracted by the District Health Information Manager did not include the TB module, which captures detailed information on diagnostic methods. Furthermore, the study period corresponds to the early phase of implementation of the WHO diagnostic package in public health facilities. During this period, diagnostic indicators such as CrAg testing and TB TB-LAM were not routinely captured in TIER.Net.

Furthemore, patients diagnosed with drug-resistant TB—including rifampicin-resistant TB (RR-TB), multidrug-resistant TB (MDR-TB), and other forms of drug-resistant TB —are registered in a separate electronic database (EDRWeb), rather than in TIER.Net. We were unable to triangulate data across these different databases, as ethical approval for this study was granted solely for the use of TIER.Net data.

Comment 4

It was good to see that the people receiving test and treat services did well overall. Could the authors comment on whether the concept of ‘starter pack’ model was used?

Response

We thank the reviewer for this insightful comment. The “starter pack” model, as described in some settings (e.g., Malawi), is not a formally adopted or nationally recognized model within the South African HIV programme. In the South African context, ART initiation under the Universal Test and Treat (UTT) strategy follows standardized national treatment guidelines, which include provision of an initial supply of first-line antiretroviral therapy at the time of initiation.

While facilities may provide an initial pre-packaged supply of ART at treatment initiation, this is implemented as part of routine guideline-based care rather than as a distinct “starter pack” model. Therefore, the outcomes observed in our study reflect implementation of the national UTT policy and standardized ART initiation protocols, rather than a specific starter pack approach

Comment 5

Could the authors comment on any impact on IRIS on retention – if not, that could go into the limitation.

Response

We thank the reviewer for this insightful comment. We acknowledge lack of data on the IRIS as one of the limitations of our study as shown in line 346 and 347 and this reads as follow “Additionally, we were unable to evaluate the impact of immune reconstitution inflammatory syndrome (IRIS) on mortality outcomes due to data limitations.”

Comment 6

Line 308 “Nevertheless” is repeated

Response

We thank the reviewer for highlighting this repetition. The duplicated word has been removed accordingly as shown in line 310.

Comment 7

Line 335 cotrimoxazole is misspelled

Response

We appreciate the reviewer for identifying this error. The spelling has been corrected, as reflected in line 337.

Comment 8

Line 343 “These” repeated

Response

We thank the reviewer for highlighting this repetition. The duplicated word has been removed accordingly as shown in line 345.

Attachment Submitted filename: Response to Reviewers-21 March 2026.docx

# Peer review: Decision Letter 1

**DOI:** 10.1371/journal.pone.0360657.r003

**Authors:** Okikiolu Badejo

13 Apr 2026

Dear Dr. Kitenge,

Thank you for submitting your manuscript to PLOS ONE. After careful consideration, we feel that it has merit but does not fully meet PLOS ONE’s publication criteria as it currently stands. Therefore, we invite you to submit a revised version of the manuscript that addresses the points raised during the review process.

Please submit your revised manuscript by May 28 2026 11:59PM. If you will need more time than this to complete your revisions, please reply to this message or contact the journal office at plosone@plos.org. When you're ready to submit your revision, log on to https://www.editorialmanager.com/pone/ and select the 'Submissions Needing Revision' folder to locate your manuscript file.

- A letter that responds to each point raised by the academic editor and reviewer(s). You should upload this letter as a separate file labeled 'Response to Reviewers'.
- A marked-up copy of your manuscript that highlights changes made to the original version. You should upload this as a separate file labeled 'Revised Manuscript with Track Changes'.
- An unmarked version of your revised paper without tracked changes. You should upload this as a separate file labeled 'Manuscript'.

If you would like to make changes to your financial disclosure, please include your updated statement in your cover letter. Guidelines for resubmitting your figure files are available below the reviewer comments at the end of this letter.

If applicable, we recommend that you deposit your laboratory protocols in protocols.io to enhance the reproducibility of your results. Protocols.io assigns your protocol its own identifier (DOI) so that it can be cited independently in the future. For instructions see: https://journals.plos.org/plosone/s/submission-guidelines#loc-laboratory-protocols. Additionally, PLOS ONE offers an option for publishing peer-reviewed Lab Protocol articles, which describe protocols hosted on protocols.io. Read more information on sharing protocols at https://plos.org/protocols?utm_medium=editorial-email&utm_source=authorletters&utm_campaign=protocols.

As the corresponding author, your ORCID iD is verified in the submission system and will appear in the published article. PLOS supports the use of ORCID, and we encourage all coauthors to register for an ORCID iD and use it as well. Please encourage your coauthors to verify their ORCID iD within the submission system before final acceptance, as unverified ORCID iDs will not appear in the published article. *Only*  the individual author can complete the verification step; PLOS staff *cannot*  verify ORCID iDs on behalf of authors.

We look forward to receiving your revised manuscript.

Kind regards,

Okikiolu Badejo, M.D., Ph.D

Academic Editor

PLOS One

Journal Requirements:

If the reviewer comments include a recommendation to cite specific previously published works, please review and evaluate these publications to determine whether they are relevant and should be cited. There is no requirement to cite these works unless the editor has indicated otherwise.

Please review your reference list to ensure that it is complete and correct. If you have cited papers that have been retracted, please include the rationale for doing so in the manuscript text, or remove these references and replace them with relevant current references. Any changes to the reference list should be mentioned in the rebuttal letter that accompanies your revised manuscript. If you need to cite a retracted article, indicate the article’s retracted status in the References list and also include a citation and full reference for the retraction notice.

Additional Editor Comments:

One reviewer has noted that their comments were not responded to. Authors are encouraged to respond to all reviewer comments. The outstanding comments appear below:

This publication adds a significant contribution to the body of work on advanced HIV disease in South Africa especially for rural South Africa. It covered data from 2008 to 2020 and highlighted better retention amongst those with Advanced HIV Disease who initiated art in the universal test and treat era including lower mortality and better viral load suppression. The study also highlighted gaps in viral load completion.

General comments: please review spelling in document including figures and tables.

Results

1. Line 184: Missing a “1) for the first time period: 2008-2011

2. Line 184: The start year for UTT period is incorrectly labelled as 2013 instead of 2016 as noted in Other parts of the manuscript including table 1.

3. Lines 222-229: The numbers for the adjusted Hazard ratios and 95% presented in the manuscript differ from the numbers shown on Table 3. Please review numbers and statements to ensure alignment with Table 3.

a. For example, The author states that patients with lower CD4 had a 15% higher sub distribution of LTFU

however table three, line 564 shows that those with a higher CD4 of 100 to 199 had a 13% higher sub distribution hazard of LTFU.

b. For certain variables the author referenced the crude hazard ratio while for others, referenced the adjusted hazard ratio. I could recommend presenting the adjusted results.

4. Lines 235-236: Finally, patients who initiated ART with a lower CD4 count of 0-99 cells/μL experienced a lower cumulative incidence of LTFU compared to with a CD4 count of 100-199 cells/μL (appendix Figure1).

a. This statement is not supported by the data in Appendix fig 1 which shows a higher cumulative incidence of LTFU for those with CD4 of 0-99 cells/μL

5. Lines 238-239: Additionally, patients receiving TB treatment exhibited a lower cumulative incidence of LTFU compared to those not receiving TB treatment, according to Figure 2.

a. This statement is not supported by the data shown on Table 3. According to that table 3 patients not on TB treatment had a lower hazards of LTFU. Recommend double checking reference groups.

b. Please clarify the denominator for this statement? Is this among the total number of patients with AHD or among those with confirmed TB? If the denominator is among those with confirmed TB who are eligible for treatment, then your findings align with the fact that patients who are engaged in TB treatment are less likely to be LTFU.

6. Line 237: “Similarly, patients enrolled in HIV care during the UTT had a lower cumulative incidence of LTFU compared to those enrolled between 2008 and 2012.”

a. This should be 2008-2011 Based on the time period Specified in the methods and table one, instead of 2008-2012 per manuscript.

7. Table 4: missing column total for the Ns for the three time periods.

Discussion

1. Given the high prevalence of TB in PLHIV in South Africa, it is important to address in the discussion some possible reasons why those not receiving TB treatment had higher retention (lines 218-219) and a significantly lower sub distribution hazard of LTFU than those receiving TB treatment (line 225-227).

[Note: HTML markup is below. Please do not edit.]

Reviewers' comments:

Reviewer's Responses to Questions

**Comments to the Author**

Reviewer #1: All comments have been addressed

Reviewer #2: (No Response)

**********

2. Is the manuscript technically sound, and do the data support the conclusions?

Reviewer #1: Yes

Reviewer #2: Yes

**********

3. Has the statistical analysis been performed appropriately and rigorously?

Reviewer #1: Yes

Reviewer #2: Yes

**********

4. Have the authors made all data underlying the findings in their manuscript fully available?

The PLOS Data policy

Reviewer #1: No

Reviewer #2: Yes

**********

5. Is the manuscript presented in an intelligible fashion and written in standard English?

Reviewer #1: Yes

Reviewer #2: Yes

**********

Reviewer #1: I didn't review a tracked changes version but the responses to my suggestions are sufficient and complete.

Reviewer #2: My comments were not addressed by the authors. I see that one reviewer's comments were addressed but my comments were not addressed.

**********

what does this mean?). If published, this will include your full peer review and any attached files.

If you choose “no”, your identity will remain anonymous but your review may still be made public.

**Do you want your identity to be public for this peer review?**  For information about this choice, including consent withdrawal, please see our Privacy Policy

Reviewer #1: No

Reviewer #2: No

**********

[NOTE: If reviewer comments were submitted as an attachment file, they will be attached to this email and accessible via the submission site. Please log into your account, locate the manuscript record, and check for the action link "View Attachments". If this link does not appear, there are no attachment files.]

To ensure your figures meet our technical requirements, please review our figure guidelines: https://journals.plos.org/plosone/s/figures

You may also use PLOS’s free figure tool, NAAS, to help you prepare publication quality figures: https://journals.plos.org/plosone/s/figures#loc-tools-for-figure-preparation.

NAAS will assess whether your figures meet our technical requirements by comparing each figure against our figure specifications.

# Author comment: Author response to Decision Letter 2

**DOI:** 10.1371/journal.pone.0360657.r004

29 Jun 2026

Dear reviewers,

Re: Responses to reviewers’ comments

Please find below a point-by-point response to the issues raised by the reviewers. The changes made to the manuscript are highlighted in track changes in the revised version.

Original Title: Evaluating Long-Term Outcomes of Patients with Advanced HIV Disease: Insights from 12 Years of Antiretroviral Therapy Scale-Up in Rural KwaZulu-Natal.

Overall impression

This is a nicely This publication adds a significant contribution to the body of work on advanced HIV disease in South Africa, especially for rural South Africa. It covered data from 2008 to 2020 and highlighted better retention amongst those with Advanced HIV Disease who initiated art in the universal test and treat era, including lower mortality and better viral load suppression. The study also highlighted gaps in viral load completion. General comments, please review spelling in the document, including figures and tables.

Comment 1

Line 184: Missing a “1) for the first time period: 2008-2011

Response

Thank you for this important comment. We have addressed the comment as shown 185-186 in line and this reads as follows “Of these 3,420 (35.6%) initiated ART during the 2008-2011 period, 3,761 (39.1%) initiated ART during 2012 and 2015, and 2,427 (49.3%) initiated during the Universal Test and Treat (UTT) (2016-2020)”

Comment 2

Line 184: The start year for UTT period is incorrectly labelled as 2013 instead of 2016 as noted in other parts of the manuscript including table 1.

Response

Thank you for this important comment. We have made changes accordingly as shown in line 186.

Comment 3

Lines 222-229: The numbers for the adjusted Hazard ratios and 95% presented in the manuscript differ from the numbers shown on Table 3. Please review numbers and statements to ensure alignment with Table 3.

a. For example, The author states that patients with lower CD4 had a 15% higher sub distribution of LTFU

however table three, line 564 shows that those with a higher CD4 of 100 to 199 had a 13% higher sub distribution hazard of LTFU.

b. For certain variables the author referenced the crude hazard ratio while for others, referenced the adjusted hazard ratio. I could recommend presenting the adjusted results.

Response

We appreciate the reviewer's careful observation. We have corrected the discrepancies between the effect estimates and 95% confidence intervals reported in the text and those presented in Table 3. The revised text now aligns with Table 3, and these changes have been made between lines 225 and 238. Additionally, to improve consistency and avoid confusion, we have presented only the adjusted effect estimates throughout the manuscript.

Comment 4

Lines 235-236: Finally, patients who initiated ART with a lower CD4 count of 0-99 cells/μL experienced a lower cumulative incidence of LTFU compared to with a CD4 count of 100-199 cells/μL (appendix Figure1).

a. This statement is not supported by the data in Appendix fig 1 which shows a higher cumulative incidence of LTFU for those with CD4 of 0-99 cells/μL

Response

We thank the reviewer for this insightful comment. We have revised the manuscript accordingly. The relevant changes have been incorporated in line 239 and now read as follows: “Finally, patients who initiated ART with a lower CD4 count of 0-99 cells/μL experienced a higher cumulative incidence of LTFU compared to with a CD4 count of 100-199 cells/μL (appendix Figure 1).”

Comment 5

Lines 238-239: Additionally, patients receiving TB treatment exhibited a lower cumulative incidence of LTFU compared to those not receiving TB treatment, according to Figure 2.

a. This statement is not supported by the data shown on Table 3. According to that table 3 patients not on TB treatment had a lower hazards of LTFU. Recommend double checking reference groups.

b. Please clarify the denominator for this statement? Is this among the total number of patients with AHD or among those with confirmed TB? If the denominator is among those with confirmed TB who are eligible for treatment, then your findings align with the fact that patients who are engaged in TB treatment are less likely to be LTFU.

Response

We thank the reviewer for this insightful comment. We have revised the manuscript accordingly. The relevant changes have been incorporated in line 242-244 and now read as follows: “Additionally, patients with TB exhibited a higher cumulative incidence of LTFU compared to those without TB. However, there was no difference in cumulative incidence of LTFU between women and men.”

We appreciate the reviewer's careful observation. We have corrected this throughout the manuscript. Specifically, of the 9,608 patients with advanced HIV disease (AHD), 630 (6.6%) had TB at the time of ART initiation. This has been corrected in line 190 and now reads: "Additionally, 6.6% of patients had TB at the time of ART initiation.”. To improve clarity and ensure consistent terminology, we have replaced references to "TB treatment" with "patients with TB" where appropriate. These revisions have been made in lines 190–191, 222–223, 229–231, 242–243, and 352–353.

Comment 6

Line Line 237: “Similarly, patients enrolled in HIV care during the UTT had a lower cumulative incidence of LTFU compared to those enrolled between 2008 and 2012.”

a. This should be 2008-2011 Based on the time period Specified in the methods and table one, instead of 2008-2012 per manuscript.

Response

We thank the reviewer for highlighting these inconsistencies. We have corrected them accordingly. The revised text has been incorporated in lines 241–243 and now reads as follows “Similarly, patients enrolled in HIV care during the UTT had a lower cumulative incidence of LTFU compared to those enrolled between 2008 and 2011”.

Comment 7

Table 4: missing column total for the Ns for the three time periods.

Response

We appreciate the reviewer's comment. We have included the missing total (N) column for each of the three time periods, as requested. These revisions have been incorporated in Table 4 and are reflected in lines 648–650.

Comment 8

Given the high prevalence of TB in PLHIV in South Africa, it is important to address in the discussion some possible reasons why those not receiving TB treatment had higher retention (lines 218-219) and a significantly lower sub-distribution hazard of LTFU than those receiving TB treatment (line 225-227).

Response

We thank the reviewer for this valuable suggestion. We have added a paragraph discussing the association between tuberculosis (TB) at ART initiation and retention in care. This addition has been incorporated in lines 389–395 and now reads as follows: “Our analysis also demonstrated that patients with AHD who had TB at the time of ART initiation were at a higher risk of LTFU than those without TB. This finding is consistent with previous studies (41, 42) showing that individuals with AHD and TB experience poorer retention in care, largely due to the increased treatment burden associated with concurrent HIV and TB therapy, debilitating medication side effects, psychosocial barriers such as stigma and fear of disclosure, food insecurity, and the financial costs of frequent clinic visits, all of which can hinder sustained engagement in HIV care.”.

Attachment Submitted filename: Response to Reviewers-26 June 2026.docx

# Peer review: Decision Letter 2

**DOI:** 10.1371/journal.pone.0360657.r005

**Authors:** Katrien Janin

1 Sep 2026

Dear Dr. Kitenge,

Thank you for submitting your manuscript to PLOS One. After careful consideration, we feel that it has merit but does not fully meet PLOS One’s publication criteria as it currently stands. Therefore, we invite you to submit a revised version of the manuscript that addresses the points raised during the review process.

Please submit your revised manuscript by Oct 16 2026 11:59PM. If you will need more time than this to complete your revisions, please reply to this message or contact the journal office at plosone@plos.org. When you're ready to submit your revision, log on to https://www.editorialmanager.com/pone/ and select the 'Submissions Needing Revision' folder to locate your manuscript file.

- A letter that responds to each point raised by the academic editor and reviewer(s). You should upload this letter as a separate file labeled 'Response to Reviewers'.
- A marked-up copy of your manuscript that highlights changes made to the original version. You should upload this as a separate file labeled 'Revised Manuscript with Track Changes'.
- An unmarked version of your revised paper without tracked changes. You should upload this as a separate file labeled 'Manuscript'.

If you would like to make changes to your financial disclosure, please include your updated statement in your cover letter. Guidelines for resubmitting your figure files are available below the reviewer comments at the end of this letter.

If applicable, we recommend that you deposit your laboratory protocols in protocols.io to enhance the reproducibility of your results. Protocols.io assigns your protocol its own identifier (DOI) so that it can be cited independently in the future. For instructions see: https://journals.plos.org/plosone/s/submission-guidelines#loc-laboratory-protocols. Additionally, PLOS One offers an option for publishing peer-reviewed Lab Protocol articles, which describe protocols hosted on protocols.io. Read more information on sharing protocols at https://plos.org/protocols?utm_medium=editorial-email&utm_source=authorletters&utm_campaign=protocols.

As the corresponding author, your ORCID iD is verified in the submission system and will appear in the published article. PLOS supports the use of ORCID, and we encourage all coauthors to register for an ORCID iD and use it as well. Please encourage your coauthors to verify their ORCID iD within the submission system before final acceptance, as unverified ORCID iDs will not appear in the published article. *Only*  the individual author can complete the verification step; PLOS staff *cannot*  verify ORCID iDs on behalf of authors.

We look forward to receiving your revised manuscript.

Kind regards,

Katrien G. Janin, PhD

Staff Editor

PLOS One

Journal Requirements:

If the reviewer comments include a recommendation to cite specific previously published works, please review and evaluate these publications to determine whether they are relevant and should be cited. There is no requirement to cite these works unless the editor has indicated otherwise.

Please review your reference list to ensure that it is complete and correct. If you have cited papers that have been retracted, please include the rationale for doing so in the manuscript text, or remove these references and replace them with relevant current references. Any changes to the reference list should be mentioned in the rebuttal letter that accompanies your revised manuscript. If you need to cite a retracted article, indicate the article’s retracted status in the References list and also include a citation and full reference for the retraction notice.

[Note: HTML markup is below. Please do not edit.]

Reviewers' comments:

Reviewer's Responses to Questions

**Comments to the Author**

Reviewer #2: (No Response)

**********

2. Is the manuscript technically sound, and do the data support the conclusions?

Reviewer #2: Yes

**********

3. Has the statistical analysis been performed appropriately and rigorously?

Reviewer #2: Yes

**********

4. Have the authors made all data underlying the findings in their manuscript fully available?

The PLOS Data policy

Reviewer #2: Yes

**********

5. Is the manuscript presented in an intelligible fashion and written in standard English?

Reviewer #2: Yes

**********

Reviewer #2: Thank you for addressing my comments.

1. The authors made updates to the TB results and noted in their response: "To improve clarity and ensure consistent

terminology, we have replaced references to "TB treatment" with "patients with TB" where appropriate." Changes were made to the text but TB treatment is still referenced on Table 3 and Fig 2 instead of TB status at ART initiation. (legend labels).

2. Fig 3 panel D: retention is misspelled.

3. Line 344, Cotrimoxazole is still misspelled as "contimoxazole".

4. Reviewer 1 inquired about the ART regimen; since Table 3 and 5 show the backbone (TDF+FTC/3TC), a footnote should be added clarifying the remaining ART regimen (EFV or DTG as indicated ) since the subheading states (ART regimen) and gives the impression that patients were treated with 2 drug combinations.

**********

what does this mean?). If published, this will include your full peer review and any attached files.

If you choose “no”, your identity will remain anonymous but your review may still be made public.

**Do you want your identity to be public for this peer review?**  For information about this choice, including consent withdrawal, please see our Privacy Policy

Reviewer #2: No

**********

[NOTE: If reviewer comments were submitted as an attachment file, they will be attached to this email and accessible via the submission site. Please log into your account, locate the manuscript record, and check for the action link "View Attachments". If this link does not appear, there are no attachment files.]

To ensure your figures meet our technical requirements, please review our figure guidelines: https://journals.plos.org/plosone/s/figures

You may also use PLOS’s free figure tool, NAAS, to help you prepare publication quality figures: https://journals.plos.org/plosone/s/figures#loc-tools-for-figure-preparation.

NAAS will assess whether your figures meet our technical requirements by comparing each figure against our figure specifications.

# Author comment: Author response to Decision Letter 3

**DOI:** 10.1371/journal.pone.0360657.r006

6 Sep 2026

04 September 2026

Dear reviewers,

Re: Responses to reviewers’ comments

Please find below a point-by-point response to the issues raised by the reviewers. The changes made to the manuscript are highlighted in track changes in the revised version.

Original Title: Evaluating Long-Term Outcomes of Patients with Advanced HIV Disease: Insights from 12 Years of Antiretroviral Therapy Scale-Up in Rural KwaZulu-Natal.

Comment 1

The authors made updates to the TB results and noted in their response: "To improve clarity and ensure consistent terminology, we have replaced references to "TB treatment" with "patients with TB" where appropriate." Changes were made to the text, but TB treatment is still referenced on Table 3 and Fig 2 instead of TB status at ART initiation. (legend labels).

Response

We appreciate the reviewer for the careful observation. We have revised the manuscript accordingly. The relevant changes have been incorporated in line 562 or Table 3 and line 558 or Figure 2.

Comment 2

Fig panel D: retention is misspelled.

Response

We appreciate the reviewer one more time for the careful observation. We have revised this as shown in line 558 or Figure 2, panel D.

Comment 3

Line 344, Cotrimoxazole is still misspelled as "contimoxazole".

Response

We thank the reviewer for this insightful comment. We have revised the manuscript accordingly. The relevant change has been incorporated in line 341-342 and now read as follows: “such as cotrimoxazole prophylaxis, fluconazole pre-emptive therapy and the presence of opportunistic infections beyond TB”.

Comment 3

Reviewer 1 inquired about the ART regimen; since Table 3 and 5 show the backbone (TDF+FTC/3TC), a footnote should be added clarifying the remaining ART regimen (EFV or DTG as indicated ) since the subheading states (ART regimen) and gives the impression that patients were treated with 2 drug combinations.

Response

Thank you for this important observation. We agree that presenting only TDF + FTC/3TC under the ART regimen heading may give the impression that participants were receiving a two-drug combination. We have therefore revised Tables 3 and 5 to clarify that TDF + FTC/3TC represented the NRTI backbone, with EFV as the third antiretroviral agent. The complete regimen is therefore presented as TDF + FTC/3TC + EFV, which reflects the use of either FTC or 3TC together with TDF and EFV.

During the study period, dolutegravir (DTG) had not yet been widely implemented across South African public health facilities, and EFV-based regimens remained commonly used. We have therefore retained EFV as the third agent in the description of the ART regimen to accurately reflect treatment practice during the study period. These changes are shown in table 1, table 3 and table 5.

Attachment Submitted filename: Response to Reviewers-04-09-2026.docx

# Peer review: Decision Letter 3

**DOI:** 10.1371/journal.pone.0360657.r007

**Authors:** Nicky McCreesh

29 Sep 2026

Evaluating Long-Term Outcomes of Patients with Advanced HIV Disease: Insights from 12 Years of Antiretroviral Therapy Scale-Up in Rural KwaZulu-Natal

PONE-D-25-51412R3

Dear Dr. Kitenge,

We’re pleased to inform you that your manuscript has been judged scientifically suitable for publication and will be formally accepted for publication once it meets all outstanding technical requirements.

Within one week, you’ll receive an e-mail detailing the required amendments. When these have been addressed, you’ll receive a formal acceptance letter and your manuscript will be scheduled for publication.

An invoice will be generated when your article is formally accepted. Please note, if your institution has a publishing partnership with PLOS and your article meets the relevant criteria, all or part of your publication costs will be covered. Please make sure your user information is up-to-date by logging into Editorial Manager at Editorial Manager® and clicking the ‘Update My Information' link at the top of the page. For questions related to billing, please contact billing support.

If your institution or institutions have a press office, please notify them about your upcoming paper to help maximize its impact. If they’ll be preparing press materials, please inform our press team as soon as possible -- no later than 48 hours after receiving the formal acceptance. Your manuscript will remain under strict press embargo until 2 pm Eastern Time on the date of publication. For more information, please contact onepress@plos.org.

Kind regards,

Nicky McCreesh

Academic Editor

PLOS One

Additional Editor Comments (optional):

Reviewers' comments:

Reviewer's Responses to Questions

**Comments to the Author**

Reviewer #2: All comments have been addressed

**********

2. Is the manuscript technically sound, and do the data support the conclusions?

Reviewer #2: Yes

**********

3. Has the statistical analysis been performed appropriately and rigorously?

Reviewer #2: Yes

**********

4. Have the authors made all data underlying the findings in their manuscript fully available?

The PLOS Data policy

Reviewer #2: Yes

**********

5. Is the manuscript presented in an intelligible fashion and written in standard English?

Reviewer #2: Yes

**********

Reviewer #2: I have no concerns about dual publication, research ethics or publication ethics. The authors have clearly addressed my comments from the last version.

**********

what does this mean?). If published, this will include your full peer review and any attached files.

If you choose “no”, your identity will remain anonymous but your review may still be made public.

**Do you want your identity to be public for this peer review?**  For information about this choice, including consent withdrawal, please see our Privacy Policy

Reviewer #2: No

**********

# Editor report: Acceptance letter

**DOI:** 10.1371/journal.pone.0360657.r008

**Authors:** Nicky McCreesh

PONE-D-25-51412R3

PLOS One

Dear Dr. Kitenge,

I'm pleased to inform you that your manuscript has been deemed suitable for publication in PLOS One. Congratulations! Your manuscript is now being handed over to our production team.

At this stage, our production department will prepare your paper for publication. This includes ensuring the following:

* All references, tables, and figures are properly cited

* All relevant supporting information is included in the manuscript submission,

* There are no issues that prevent the paper from being properly typeset

You will receive further instructions from the production team, including instructions on how to review your proof when it is ready. Please keep in mind that we are working through a large volume of accepted articles, so please give us a few days to review your paper and let you know the next and final steps.

Lastly, if your institution or institutions have a press office, please let them know about your upcoming paper now to help maximize its impact. If they'll be preparing press materials, please inform our press team within the next 48 hours. Your manuscript will remain under strict press embargo until 2 pm Eastern Time on the date of publication. For more information, please contact onepress@plos.org.

You will receive an invoice from PLOS for your publication fee after your manuscript has reached the completed accept phase. If you receive an email requesting payment before acceptance or for any other service, this may be a phishing scheme. Learn how to identify phishing emails and protect your accounts at https://explore.plos.org/phishing.

If we can help with anything else, please email us at customercare@plos.org.

Thank you for submitting your work to PLOS One and supporting open access.

Kind regards,

PLOS One Editorial Office Staff

on behalf of

Dr. Nicky McCreesh

Academic Editor

PLOS One
